# BIO-CXRNET: a robust multimodal stacking machine learning technique for mortality risk prediction of COVID-19 patients using chest X-ray images and clinical data

**DOI:** 10.1007/s00521-023-08606-w

**Published:** 2023-05-04

**Authors:** Tawsifur Rahman, Muhammad E. H. Chowdhury, Amith Khandakar, Zaid Bin Mahbub, Md Sakib Abrar Hossain, Abraham Alhatou, Eynas Abdalla, Sreekumar Muthiyal, Khandaker Farzana Islam, Saad Bin Abul Kashem, Muhammad Salman Khan, Susu M. Zughaier, Maqsud Hossain

**Affiliations:** 1 Department of Electrical Engineering, Qatar University, P.O. Box 2713, Doha, Qatar; 2 Department of Physics and Mathematics, North South University, Dhaka, 1229 Bangladesh; 3 NSU Genome Research Institute (NGRI), North South University, Dhaka, 1229 Bangladesh; 4 Department of Biology, University of South Carolina (USC), Columbia, SC 29208 USA; 5 Anesthesia Department, Hamad General Hospital, P.O. Box 3050, Doha, Qatar; 6 Department of Radiology, Hamad General Hospital, P.O. Box 3050, Doha, Qatar; 7 Department of Computer Science, AFG College with the University of Aberdeen, Doha, Qatar; 8 Department of Basic Medical Sciences, College of Medicine, QU Health, Qatar University, P.O. Box 2713, Doha, Qatar

**Keywords:** Multimodal system, COVID-19, Clinical data, Chest X-ray, Prognostic model, Deep learning, Classical machine learning

## Abstract

Nowadays, quick, and accurate diagnosis of COVID-19 is a pressing need. This study presents a multimodal system to meet this need. The presented system employs a machine learning module that learns the required knowledge from the datasets collected from 930 COVID-19 patients hospitalized in Italy during the first wave of COVID-19 (March–June 2020). The dataset consists of twenty-five biomarkers from electronic health record and Chest X-ray (CXR) images. It is found that the system can diagnose low- or high-risk patients with an accuracy, sensitivity, and *F*1-score of 89.03%, 90.44%, and 89.03%, respectively. The system exhibits 6% higher accuracy than the systems that employ either CXR images or biomarker data. In addition, the system can calculate the mortality risk of high-risk patients using multivariate logistic regression-based nomogram scoring technique. Interested physicians can use the presented system to predict the early mortality risks of COVID-19 patients using the web-link: Covid-severity-grading-AI. In this case, a physician needs to input the following information: CXR image file, Lactate Dehydrogenase (LDH), Oxygen Saturation (O_2_%), White Blood Cells Count, C-reactive protein, and Age. This way, this study contributes to the management of COVID-19 patients by predicting early mortality risk.

**Supplementary Information:**

The online version contains supplementary material available at 10.1007/s00521-023-08606-w.

## Introduction

As of January 9, 2023 [1], the COVID-19 pandemic had caused about 6.71 million fatalities and 668 million infections, with new variations periodically developing [2]. Business, economic, and social dynamics on a worldwide scale were all affected. Governments throughout the world have adopted flight restrictions, social isolation, and heightened awareness of hygiene. COVID-19 is easily mistaken for other viral infections making detection challenging [3, 4]. Reverse-transcription polymerase chain reaction (RT-PCR) arrays are the primary approved diagnostic approach for COVID-19 identification [5–7]. Contamination/damage in the sample or viral changes to the COVID-19 genome may hinder its detection performance [8–10]. Sadly, despite the need of prompt diagnosis, this test can take up to six hours from sample collection and may require multiple tests to distinguish between false-negative and false-positive results [7, 11]. The false detection due to contamination concerns in RT-PCR detection and the delays caused have motivated several studies [12–14] have demonstrated chest computed tomography (CT) imaging as a non-invasive alternative. In addition, multiple publications [12, 13, 15] have advised a CT scan as a follow-up test for patients with COVID-19 symptoms and negative RT-PCR findings due to them being non-invasive and detailed that can aid in diagnosis. However, it has poor sensitivity in early instances of COVID-19 [16], and image gathering is time-consuming, susceptible to infection transmission (since it is performed in close proximity to patients) [17], and expensive [18]. On the other hand chest X-ray (CXR) imaging is less expensive, faster, and more widely available than computed tomography (CT), and it exposes the body to less radiation [19]. Recently chest X-rays are widely used as a COVID-19 screening alternative, and their predictive value has been established [20]. As early COVID-19 cases had bilateral, multifocal ground-glass opacities (GGO) with posterior or peripheral distribution, predominantly in the lower lung lobes, which progressed to pulmonary consolidation [21, 22]. Many lung diseases have similar symptoms. Thus, doctors struggle to differentiate between COVID-19 infection and other forms of viral pneumonia. Consequently, symptom similarities may result in a misdiagnosis, delayed treatment, or even death in this instance. Therefore, there is an urgent need for technology to assist physicians in their analysis.

Significant advances in Deep Learning approaches have led to state-of-the-art performance in a variety of Computer Vision applications, including image classification, object recognition, and image segmentation, in recent years. Because of this development, solutions based on deep learning are currently applied in a wider range of fields. Since the advent of deep Convolutional Neural Networks (CNNs), the use of CNNs to CXR images has been the topic of substantial research and broad adoption. Rajpurkar et al. [23] proposed the CheXNet network by updating Densenet121 on one of the largest Chest X-ray datasets [24] consisting of one hundred thousand X-ray pictures for fourteen distinct diseases. Rahman et al. [25] trained CXRs to detect pulmonary tuberculosis using a dataset of 3500 infected and 3,500 normal CXRs (TB). In addition, they retrained the DenseNet201 network with TB and normal datasets, attaining a TB diagnosis sensitivity of 99.57%. Khuzani et al. [26] postulated that a set of CXR image features might be built using the dimensionality reduction method to create an effective machine learning classifier capable of distinguishing COVID-19 cases from non-COVID-19 cases with high accuracy and sensitivity. Mathew et al. [27] developed a Siamese neural network-based severity score to automatically quantify radiographic COVID-19 pulmonary disease severity. This score was validated with pulmonary X-ray severity (PXS) scores from two thoracic radiologists and one radiologist-in-training. Kim et al. [28] suggested a fully automated triage pipeline that analyzes chest radiographs for the presence, severity, and progression of COVID-19 pneumonia with 79.9% accuracy. In [29], Maguolo and Nanni questioned the efficacy of COVID-19 detection from X-rays in various literature and suggested that it should incorporate larger and more diverse X-rays to eliminate biases. Robert et al. [30] have reached a similar conclusion by doing a comprehensive literature study and proposing the use of a wide and diversified dataset for the idea of COVID-19 detection from Chest X-rays. The authors of this study were also the pioneers in presenting a cutting-edge deep learning model for detecting pneumonia [31] and COVID-19 [32] from chest X-rays. However, until recently, lung segmentation was used as the first step in their detection technique [33, 34], which assisted to localize the decision-making area for machine learning networks. They generated 704 X-ray images for Normal and TB patients using the well-known Montgomery [35] and Shenzhen [36] CXR lung mask databases. In extreme COVID-19 situations, where the lungs are severely deformed, or where images are of low resolution, the segmentation performance can degrade. Using an effective human–machine collaboration technique to annotate ground-truth lung segmentation masks, another study has built the largest benchmark dataset with 33,920 CXR images and 11,956 COVID-19 samples using a human–machine collaborative strategy [37]. According to the authors’ knowledge, this is the largest CXR lung segmentation dataset, which can aid in the development of CXR-related computer-aided diagnostic tools employing deep learning techniques. In this study, the researchers segmented the lung areas from the CXR images using the model trained on this cutting-edge dataset. In a previous study [38], we examined the effect of image enhancement techniques on segmented lungs for COVID-19 prediction, confirming that gamma correction enhancement provided an F1-score of approximately 90% using a dataset of 18,479 Chest X-ray images (8851 normal, 6012 non-COVID other lung diseases, and 3616 COVID-19) and their ground truth lung masks. Huang and Liao in [39] have proposed a lightweight CNN-based network (LightEfficientNetV2) for COVID-19 detection with the help of segmented lung images. The network achieved 98.33% accuracy in COVID-19 disease from Pneumonia and Normal using 21,000 images. They claimed that the same network achieved 97.48% accuracy on CT images. Despite the benefits of radiological imaging being non-invasive and the application of machine learning speeding up the diagnosis, several studies have favored accurate blood biomarkers since variations in them can also help determine the severity, and progression of an abnormality [40, 41].

Recent research indicates that biomarkers can play a significant role in providing vital information about an individual's health and recognizing COVID-19. In addition, they can be utilized to diagnose severity, progression, and forecast mortality. Sarah et al. [42] introduced the Kuwait Progression Indicator (KPI) score as a predictor of the severity of COVID-19 progression. The KPI model was based on laboratory variables, which are objectively measurable measurements, as opposed to grading systems that rely on self-reported symptoms and other subjective features. Patients were classified as low risk if their KPI score was less than − 7 and as high risk if it was greater than 16; however, those with a score between − 6 and 15 had an unknown likelihood of advancement. This restricts its applicability to a broad range of patient populations. Weng et al. [43] presented the ANDC early prediction score to predict COVID patient mortality risk. This model was constructed using information from 301 adult individuals with laboratory-confirmed COVID-19. Age, neutrophil-to-lymphocyte ratio, D-dimer, and C-reactive protein were identified as major predictors of mortality for COVID-19 patients by LASSO regression. Area under the curve (AUC) values of 0.921 and 0.975 for the derivation and validation cohorts, respectively, indicate that the nomogram was well-calibrated and discriminative. Patients with COVID were separated into three groups based on ANDC cutoff values of 59 and 101. The low-risk group (ANDC < 59) had a mortality probability below 5%, the moderate-risk group (59 < ANDC < 101) had a mortality probability between 5 and 50%, and the high-risk group (ANDC > 101) had a mortality probability greater than 50%. Using a dataset of 444 patients, Xie et al. [44] created a predictive model that integrates age, lactate dehydrogenase (LDH), lymphocyte count, and SpO_2_ as independent predictors of death. The model performed well in both internal (*c* = 0.89) and external (*c* = 0.98) validations. However, the model over predicted low-risk individuals while under predicting high-risk people. These severity scoring can help in allocating resources efficiently to the high-risk predicted patients. Intensive care units (ICUs) are essential for preserving severely ill COVID-19 patients because they provide oxygen, 24-h monitoring, care, and when necessary, assisted ventilation. In regions with a high COVID-19 infection incidence, therefore, ICU beds are a useful resource [45–47]. Within the first hour of a hospital visit, routinely collected healthcare data such as blood tests and vital signs assessments are typically available. These data give the COVID-19 patient change patterns observed in various retrospective observational investigations [48–50]. The results of these research indicate that alanine aminotransferase (ALT), lymphocyte count, D-dimer, C-reactive protein (CRP), and bilirubin concentrations are significant clinical markers. Islam et al. in [51] developed a generic and reliable predictive model with an accuracy of 85.35% for ICU admission for COVID-19 patients using the optimal feature combination from the patient data upon admission utilizing data from the pulmonology department of Moscow City State Hospital. Significant risk variables for ICU admission were identified as C-reactive protein (CRP), chest computed tomography (CT), lung tissue damage (%), age, hospital admission, and fibrinogen parameters at hospital admission. Consequently, clinical biomarkers can be utilized to construct a highly accurate prognosis model utilizing traditional and deep learning methods.

Convolutional Neural Networks (CNNs) can be trained to classify diseases based on radiographic and other images but cannot consistently identify the underlying medical cause. Using a combination of patient symptoms, physical exam findings, laboratory data, and radiologic imaging findings, the underlying etiology and severity can be diagnosed. Consequently, machine learning algorithms that combine information from Chest X-rays with other clinical data from the electronic health record (her) will be able to better precisely predict the patient's severity. However, attempts to combine Electronic Health Record (HER) and imaging data for machine learning applications in healthcare have not been widely studied. Few studies have utilized a combination of radiographic imaging, clinical biomarker data, and artificial intelligence to predict the prognosis of COVID-19 patients. Jiao et al. in [52], using patients data from hospitals in USA, have developed a machine learning model using clinical data and CXR pictures to predict the severity and development of COVID-19 with an AUC of 82%. Chieregato et al. [53] proposed a multimodal approach based on CT images and clinical parameters, which were supplied to Boruta feature selection algorithm with ShAP (SHapley Additive exPlanations) values, and then the CatBoost gradient boosting classifier demonstrated an AUC of 0.949% for reduced features on the holdout test set. With a probability score based on the significance of SHAP features, the model aimed to provide clinical decision support to medical doctors. However, the published research has either yielded unsatisfactory results, employed tiny datasets, limiting the generalizability of the models, or employed CT, which has drawbacks as a technology. Notably, there has been research employing a DNA-based approach, but such data are not publicly accessible, and genome sequence-based investigations are computationally expensive [54].

As it can be seen that the pandemic has triggered much research in the early detection of pulmonary abnormalities using clinical imaging such as CT scans, Chest X-Rays but had some limitations, which could be only addressed from clinical examinations from blood biomarkers [55–57]. The advances in Machine Learning approaches have further catapulted the early detection automatically with high reliability and without the need of medical expert opinion, but there is a need to make it more reliable combining the imaging and blood biomarkers information. The authors of this study were inspired by the aforementioned pitfalls to create a multimodal system that uses CXR and a clinical biomarker-based system to stratify the severity of COVID-19 patients and their risk of death. Although most of the clinicians agree that there is a great need of multimodal system but the main challenge to develop such a system is the availability of such multimodal dataset. Even though a plethora of publications came out in the early and later stage of the pandemic, a very few works proposed different multimodal system to make the model reliable and explainable to the clinicians. This is one of the first studies to develop a COVID-19 severity prediction model using both CXR and biomarkers. The paper proposes a comprehensive, dependable, and novel approach that supplements all previous work in this domain. The method is applicable not only for COVID-19 detection and severity classification but also for any other lung abnormality-related complications. The following details help to explain the approach's novelty and utility:

- To segment the lungs from Chest X-rays, the authors used a robust segmentation network (which they proposed in their previous work [37]). This type of segmentation will aid the machine learning network in determining the region of interest.
- To extract features from the segmented X-rays, the cutting-edge machine learning network ChexNet [58, 59] (which was developed using the largest Chest X-ray dataset) was used.
- A multimodal technique based on Chest X-rays and Common Blood Count features was used. This will allow the network to perform more accurately when the severity cannot be determined solely by X-rays.
- Applied the stacking method to improve classification and severity performance.
- Created a nomogram scoring technique that clinicians can use to predict the severity of COVID-19 patients.
- The entire solution was implemented as an easy-to-use app for clinicians.

The rest of the article is organized as follows: Sect. 2 describes the study’s methodology, which includes dataset descriptions, preprocessing stages, machine learning and stacking techniques, and the development of a nomogram-based scoring system. Section 3 presents the experimental results and reports on the performance of the scoring technique, while Sect. 4 explains the results. Section 5 concludes the article by making future recommendations.

## Methodology

This study included two major investigations. The first study used a multimodal stacking model-based approach combining CXR images and clinical data to predict the severity risk of COVID-19 patients, while in the second study, CXR images and clinical biomarkers-based combined features were used to predict the death outcome in high-risk patients using a nomogram-based scoring system. The method is organized with the following sub-sections: System Architecture, Dataset Description, Statistical Analysis, Data Preprocessing, Experiments and Performance Metrics.

### System architecture of the proposed system

**Fig. 1:**
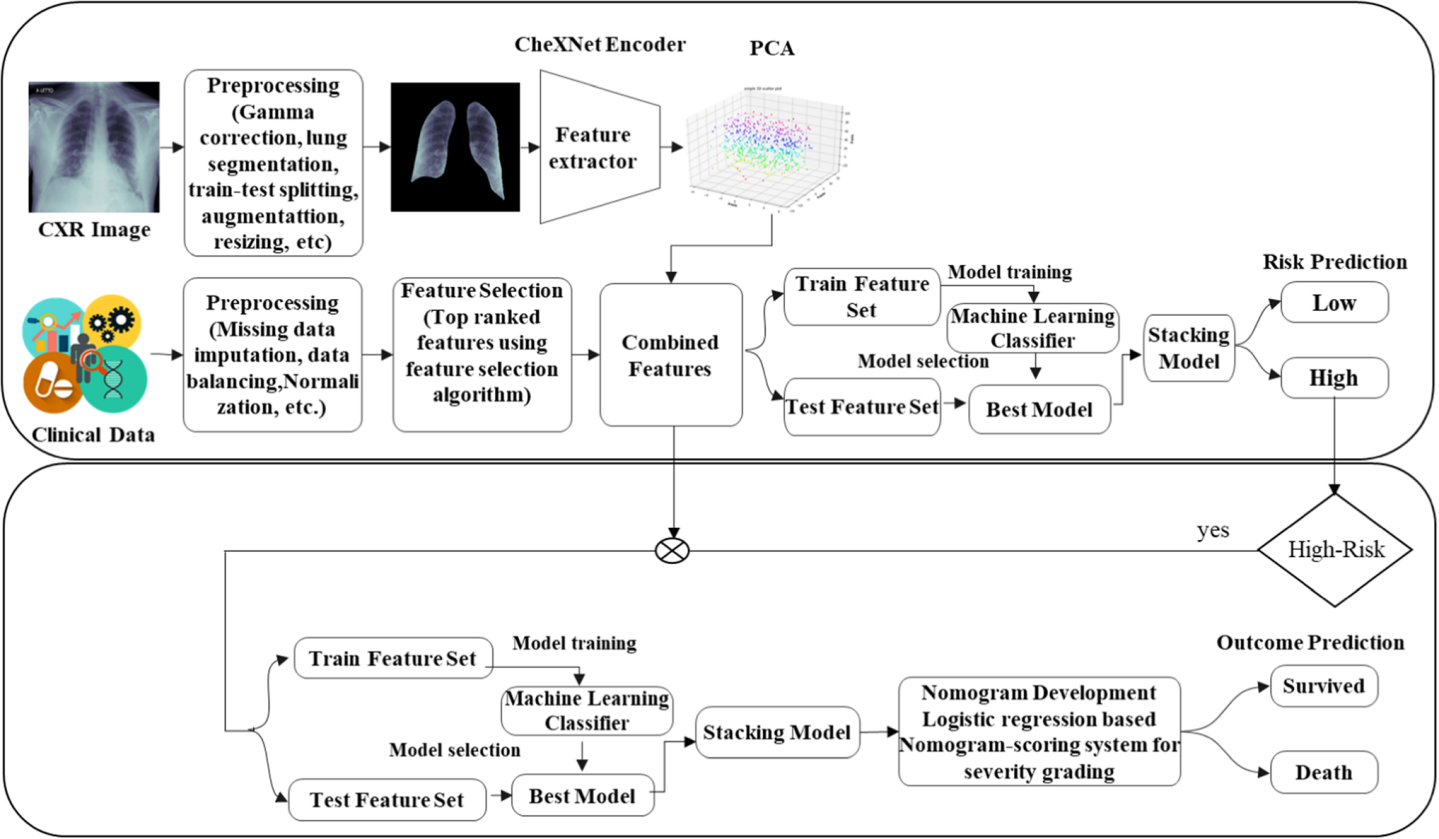
Overview of the proposed system architecture

First, CXR images are preprocessed, and the lung area is segmented and fed to a pre-trained deep CNN model to extract image features, which are then reduced in dimensionality using principal component analysis (PCA). Clinical data were processed in parallel, and clinical features were ranked using a feature selection algorithm. Finally, the PCA components and top-ranked clinical features were combined to create a stacking ensemble model to predict whether patients were low or high risk. Then the high-risk patient’s combined reduced dimensionality features were used to develop another stacking model. Furthermore, we developed a scoring technique based on a nomogram using the stacking model for the early prediction of death outcomes. The methodology is depicted schematically in Fig. 1.

**Fig. 2:**
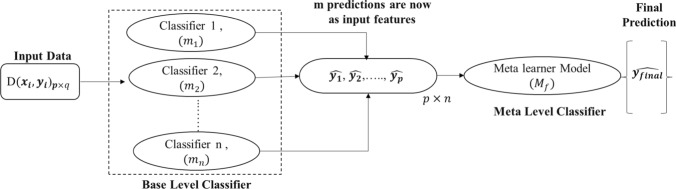
Proposed stacking model architecture

The study proposed a stacking-based approach and compared the performance with conventional ML classifiers. This approach consists of two-step learners such as base learners and meta learners. The three best-performing ML classifiers were selected as base learner models in the stacking architecture and logistic regression was used for the meta learner model ($$M_{f} )$$ in the second phase of the stacking model and finally produced the final prediction. Figure 2 shows the architecture of the proposed stacking model which combines *N* numbers of best-performing classifiers $$m_{1} , \ldots ,m_{n}$$ using an input dataset *D*, which has a feature vector ($${\varvec{x}}_{{\varvec{i}}}$$) and corresponding label ($${\varvec{y}}_{{\varvec{i}}}$$). In the first step, *n* base level ML classifier produces the prediction probabilities $$y_{1} , \ldots ,y_{p}$$. Finally, the prediction probabilities of the best performing base learners feed to a logistic regression-based meta-learner classifier ($$M_{f }$$) for the final prediction.

Each ML classifier in the base learner predicts a probability distribution according to the output class values. Therefore, a probability distribution is created for the input x using the predictions of the base-level classifier set m in Eq. 1:

1 $$ {\text{PD}}^{m} \left( x \right) = \left( {{\text{PD}}^{m} \left( {l_{1} |x} \right),{\text{PD}}^{m} \left( {l_{2} |x} \right), \ldots ,{\text{PD}}^{m} \left( {l_{r} |x} \right)} \right) $$

where $(l_{1} ,l_{2} , \ldots ,l_{r} )$ is the original class values, and ${\text{PD}}^{m} \left( {l_{i} {|}x} \right)$ denotes the probability distribution such as x belongs to a class $l_{i}$ as estimated (and predicted) by classifier m. The class $l_{k}$ with the highest-class probability ${\text{PD}}^{m} \left( {l_{i} {|}x} \right)$ is predicted by classifier m. The meta-learner attributes are the probabilities produced for each class by each of the base-level classifiers, i.e., ${\text{PD}}^{{m_{k} }} \left( {l_{i} |x} \right)$ for $i = 1, \ldots , r$ and $k = 1, \ldots , n$; where r and n are the number of classes and the number of base-level classifiers.

### Dataset description

**Fig. 3:**
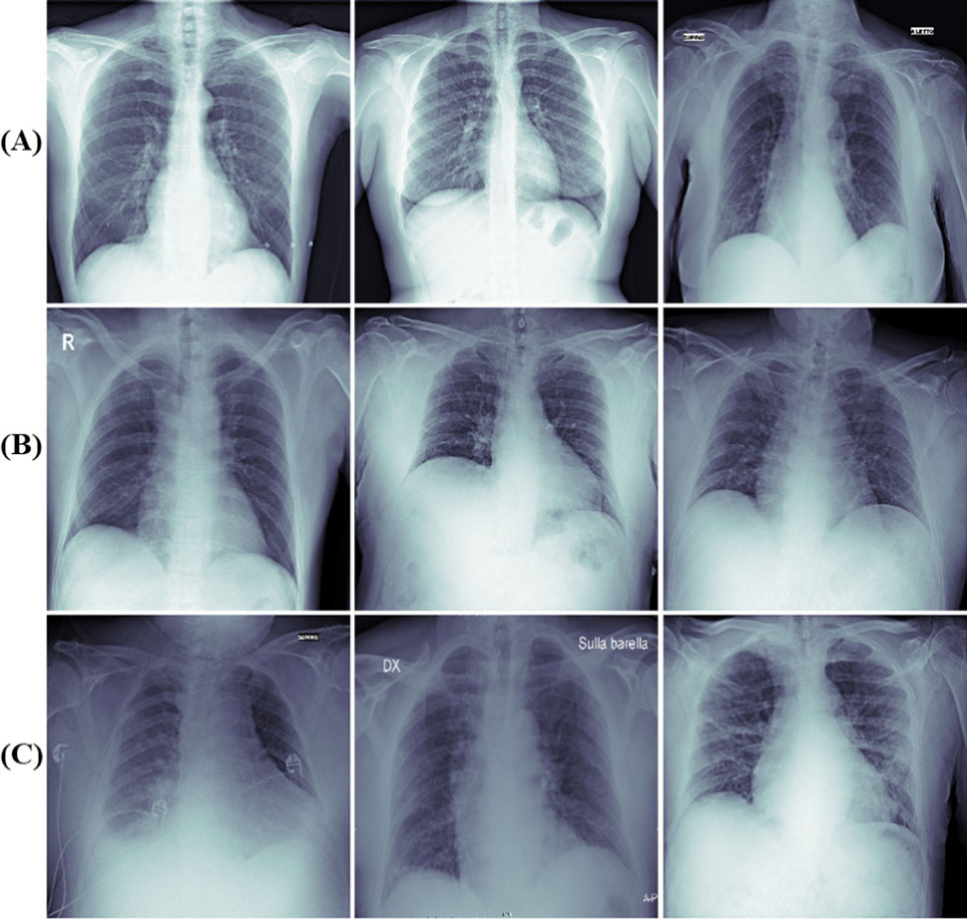
Chest X-ray sample images for COVID-19. **A** Low-risk patients, **B** High-risk patients with survival outcomes, and **C** High-risk patients with death outcomes

The study utilized a dataset from the first wave of COVID-19, collected between March and June of 2020, which contained both CXRs and clinical data collected from six Italian hospitals at the time of admission for symptomatic COVID-19 patients [60]. This dataset includes a Posterior Anterior (PA) or Anterior Posterior (AP) view of 930 X-ray images and clinical data for COVID-19-positive patients [60]. Each patient tested positive for COVID-19 using RT-PCR. This data collection includes 396 (42.6%) low-risk individuals and 534 (57.4%) high-risk patients. In addition, 364 (68.2%) of the 534 high-risk patients survived, while 170 (31.8%) perished. Figure 3 depicts CXR image samples from the dataset.

### Statistical characteristics

A statistical analysis of the patient’s demographic, signs and symptoms, clinical data, comorbidity, and the outcome was performed using Stata/MP 13.0 software. The dataset includes gender, age, and twenty-three signs and symptoms, comorbidity, and clinical biomarkers. Table 1 shows the statistical characteristics of 25 parameters (age, gender, sign and symptoms, comorbidity, clinical biomarkers). Gender is represented numerically and in percentages. For the remaining variables, the number of missing data (N), presence and absence of signs and symptoms, mean (M), and standard deviation (SD) were reported. Gender was subjected to univariate analysis (Chi-square test), while the other variables were subjected to Wilcoxon’s ranked tests. Using a 95% statistical significance criterion, the *p* value was considered significant if it was less than 0.05.

**Table 1:** Summary of statistical characteristics of the study patients

Item	Low-risk	High-risk	Total	Statistics	*P* value
Gender				*χ*^2^ = 16.45	< .05
Male (%)	159 (40%)	− 149 (28%)	308 (33%)		
Female (%)	237 (60%)	385 (72%)	622 (67%)		
Age (years)				*Z* = − 8.5	< .05
*N*	396 (0)	534 (0)	930 (0)		
*M* ± SD	60.3 ± 15.9	67.8 ± 13.5	64.7 ± 14.9		
Body temperature (°C)				*Z* = − 7.55	< .05
*N*	354 (42)	456 (78)	810 (120)		
*M* ± SD	37.5 ± 0.93	39.6 ± 1.3	37.56 ± 0.98		
Cough (%)				*Z* = − 6.67	< .05
*N*	395 (1)	530 (4)	925 (5)		
Yes/no	210/185	460/70	670/255		
Difficulty in breathing				*Z* = − 9.35	< .05
*N*	395 (1)	531 (3)	926 (4)		
Yes/no	180/215	450/81	630/296		
Red blood cell (10^9^ L)				*Z* = − 16.56	< .05
*N*	371 (25)	515 (19)	886 (44)		
*M* ± SD	4.68 ± 0.7	4.56 ± 0.71	4.59 ± 0.7		
White blood cell count (10^9^ L)				*Z* = − 8.77	< .05
*N*	386 (10)	524 (10)	910 (20)		
*M* ± SD	6.08 ± 2.76	7.87 ± 4.36	7.1 ± 3.87		
CRP (mg/dL)				*Z* = − 3.53	< .05
*N*	377 (19)	514 (20)	891 (39)		
*M* ± SD	23.01 ± 43.3	39.5 ± 69.4	32.5 ± 60.33		
Fibrinogen (mg/dL)				*Z* = 0.174	0.912
*N*	75 (321)	141 (393)	216 (714)		
*M* ± SD	561.07 ± 115	641.19 ± 172	613.37 ± 159		
Glucose (mg/dL)				*Z* = − 11.84	< .05
*N*	302 (94)	439 (95)	741 (189)		
*M* ± SD	114.5 ± 48.2	130.9 ± 60.32	124.26 ± 56		
LDH (U/L)				*Z* = − 3.28	< .05
*N*	291 (105)	404 (130)	695 (235)		
*M* ± SD	282.4 ± 114	442.4 ± 272	375.4 ± 234		
INR				*Z* = − 7.93	< .05
*N*	255 (141)	413 (121)	668 (262)		
*M* ± SD	1.15 ± 0.29	1.3 ± 0.76	1.24 ± 0.63		
D-dimer				*Z* = − 1.81	0.064
*N*	106 (290)	144 (390)	250 (680)		
*M* ± SD	1055.8 ± 1384	3780.6 ± 8635	2625.3 ± 6741		
O_2_ percentage				*Z* = − 6.42	< .05
*N*	289 (107)	365 (169)	654 (276)		
M ± SD	95.4 ± 3.72	89.5 ± 8.01	92.3 ± 7.02		
PaO_2_ (mmHg)				*Z* = − 8.33	< .05
*N*	288 (108)	412 (122)	800 (130)		
*M* ± SD	75.5 ± 17.15	69.95 ± 31.26	72.21 ± 26.5		
SaO_2_ (%)				*Z* = 1.36	0.845
*N*	165 (231)	212 (322)	377 (553)		
*M* ± SD	94.92 ± 4.38	89.95 ± 8.92	92.13 ± 7.69		
PaCO_2_ (mmHg)				*Z* = 5.36	< .05
*N*	278 (118)	403 (131)	681 (249)		
*M* ± SD	33.49 ± 5.4	33.1 ± 6.9	33.26 ± 6.34		
pH				*Z* = − 2.01	< .05
*N*	271 (125)	386 (148)	657 (273)		
*M* ± SD	7.45 ± 0.05	7.15 ± 0.06	7.35 ± 0.05		
Cardiovascular disease				*Z* = 7.78	< .05
*N*	335 (61)	467 (67)	802 (128)		
Yes/no	230/105	345/122	575/227		
Heart failure (%)				*Z* = 6.40	< .05
*N*	333 (63)	465 (69)	798 (132)		
Yes/no	215/118	288/187	503/305		
High blood pressure				*Z* = − 5.66	< .05
*N*	337 (59)	467 (67)	804 (126)		
Yes/no	227/110	330/337	557/447		
Cancer				*Z* = − 1.84	0.067
*N*	337 (59)	467 (67)	804 (126)		
Yes/no	112/225	164/303	276/528		
Chronic kidney disease (%)				*Z* = − 5.81	< .05
*N*	337 (59)	467 (67)	804 (126)		
Yes/no	156/181	320/147	476/328		
Respiratory disease				*Z* = 0.177	.865
*N*	262 (134)	290 (244)	552 (378)		
Yes/no	162/100	195/95	357/195		

### Data preprocessing

This section discusses the data preprocessing steps for both data modalities in detail.

#### Chest X-ray image preprocessing

A. Gamma correction

**Fig. 4:**
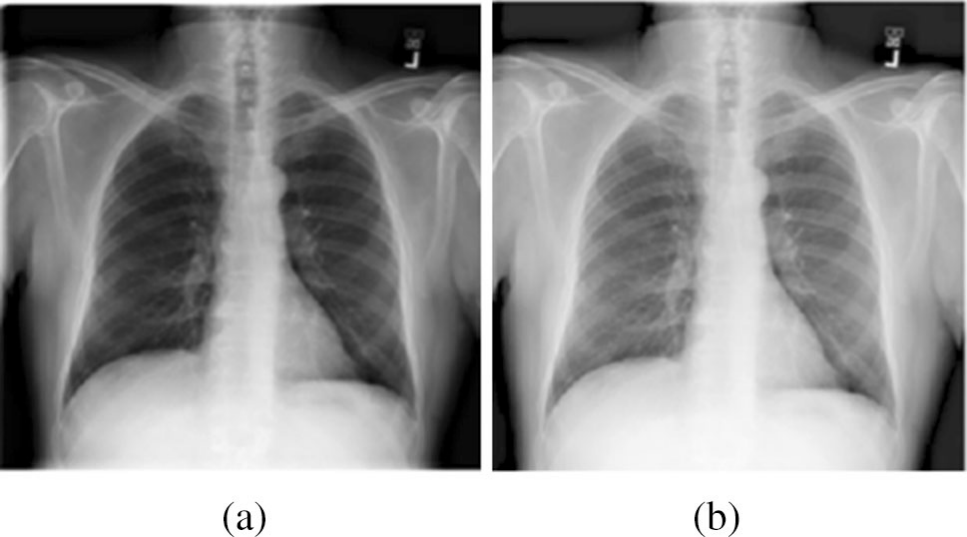
Samples X-ray image for before (**a**) and after (**b**) applying gamma correction

Image enhancement is a common image-processing technique that emphasizes important information in an image while reducing or removing other information to improve identification quality. As demonstrated in our previous work [38], gamma correction was applied to CXRs, which improves COVID detection performance by improving image quality. For image normalization, linear operations such as pixel-wise scalar multiplication, addition, and subtraction are frequently used, whereas the Gamma correction technique is a nonlinear operation performed on the pixels of the source image. Gamma correction employs a projection link with gamma and pixel values determined by the internal map. The pixel value here can range from 0 to 255. Figure 4 shows a samples X-ray image for before and after applying gamma correction. If *G* is the gray scale value, then the gamma corrected output pixel *s(G)* can be written as in Eq. (2):

2 $$ s\left( G \right) = 255\left( \frac{G}{255} \right)^{1/\gamma \left( G \right)} $$

where *γ*(*G*) represents the gamma value.

B. Lung segmentation

**Fig. 5:**
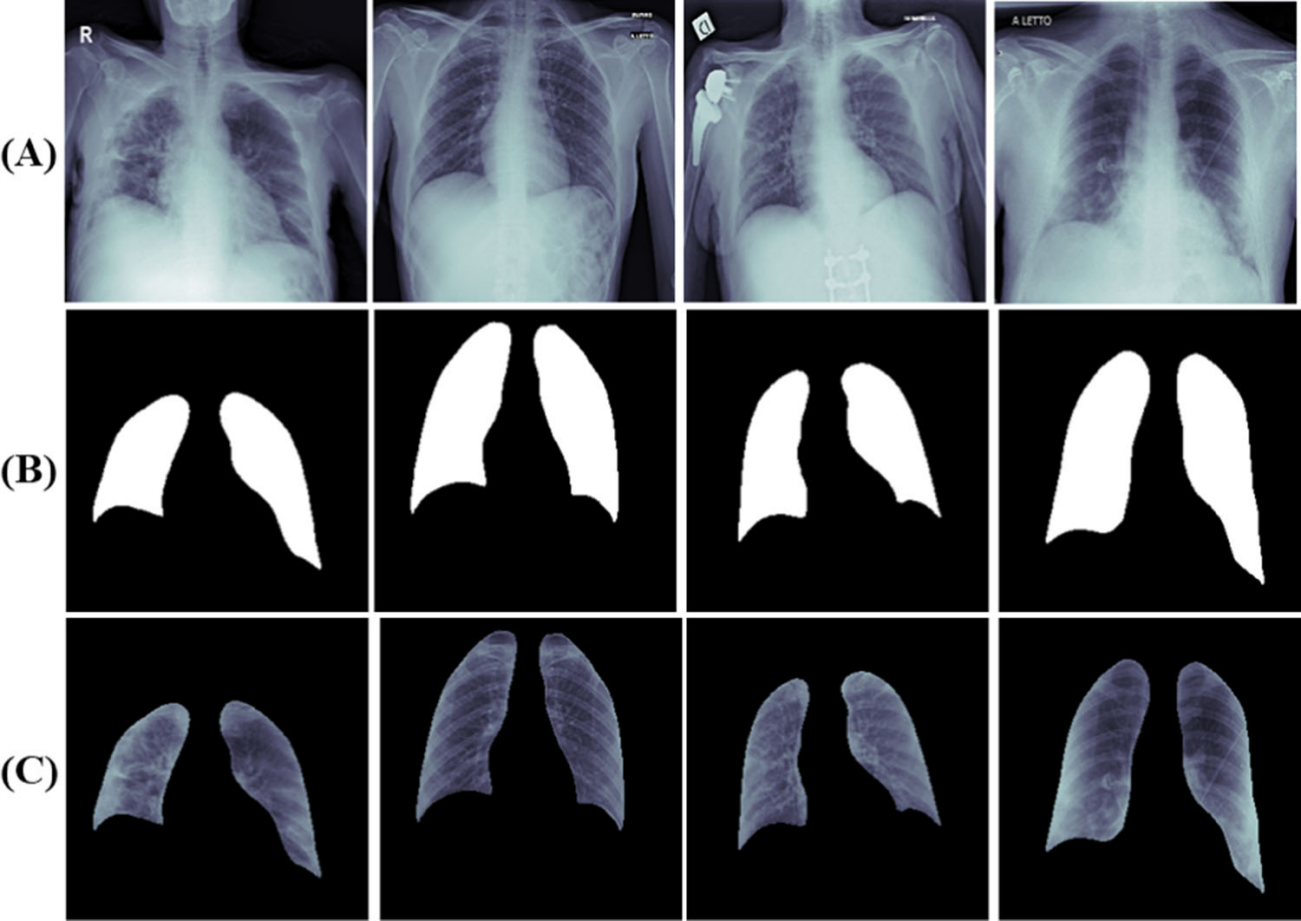
Samples X-ray images from the study dataset (**A**), generated masks by the best performing densenet121 FPN model (**B**) and corresponding segmented lung (**C**)

As previously discussed, it is critical to localize the region of interest for machine learning networks, in this case, the lungs in the Chest X-rays. In our previous work for CXR lung segmentation [37], the Feature Pyramid Networks (FPN) [61] segmentation network with the DenseNet121 [59] encoder as a backbone outperformed other conventional segmentation networks. In [37], three segmentation architectures with different encoder backbones: U-Net [62], U-Net +  + [63], and Feature Pyramid Networks (FPN) were investigated [61]. It segmented the lung area very accurately using the FPN network with DenseNet121 as the backbone, which was confirmed by experienced radiologists. Figure 5 depicts some of the X-ray images and their corresponding masks.

C. Feature extraction

To extract important features from segmented Chest X-rays, a ChexNet CNN model based on DenseNet-121 [59] architecture was used. It’s worth noting that CheXNet is a DenseNet variant that was trained on a large Chest X-ray dataset, and the pre-trained model is publicly available. As demonstrated in our previous work [38], it performed exceptionally well in the COVID-19 classification task. To extract useful features from the segmented lung area of the CXR images, features from the CheXNet model’s last layer (‘AvgPool’) before the Softmax layer were extracted.

D. PCA for dimensionality reduction

To reduce the dimensionality of the feature space produced by the ChexNet model, a feature reduction technique known as Principal Component Analysis (PCA) was used. It converts high-dimensional data into a new lower-dimensional representation with the least amount of reconstruction error. There is no redundant data in the reduced set because all the fundamental components are orthogonal to one another. Whitening was used to calculate PCA, which can improve accuracy by forcing data to meet certain assumptions.

#### Clinical data preprocessing

A. Data imputation and normalization

Missing data imputation is the most critical stage in clinical data preprocessing for machine learning model construction. Many blood biomarkers were obtained for each patient, and many of them were missing in some patients. Rather than removing missing data for the various variables, various imputation techniques were investigated. Deleting the missing variable may result in the loss of critical and contextual information, as well as having an impact on the generalized representation of the dataset [35]. Missing value imputation methods based on machine learning (ML) have grown in popularity. This technique, on the other hand, necessitates the creation of a separate model for each missing data column. In this study, a popular data imputation technique known as multivariate imputation by chained equations (MICE) was used to deal with missing data. According to the literature and previous works of the authors [64–67], the MICE technique outperforms other imputation techniques for clinical data [68].

The effectiveness of machine learning models for generalized performance is heavily dependent on the input data quality. The process of scaling or changing data so that each feature contributes equally to the training process is referred to as “data normalization.” Numerous studies have shown that normalization improves the performance of machine learning models [29]. Z-score normalization was used in this study by subtracting the average of the data and dividing it by the standard deviation.

B. Top-ranked features

The feature selection technique selects the features that have the greatest influence on the output prediction. It helps to reduce overfitting, typically improves accuracy, and drastically reduces training time. Some of the feature selection methods used in the literature include univariate selection, principal component analysis (PCA), recursive feature elimination (RFE), bagged decision trees (e.g., random forest), and boosted trees (e.g., Extreme Gradient Boosting). Random forest frequently outperforms other methods due to its ability to handle datasets with many predictor variables [69]. As a result, a random forest-based feature selection technique was used in this study to rank the features in risk prediction out of 25 variables, including age, gender, sign and symptoms, comorbidity, and clinical biomarkers.

### Experiments

All the experiments in this study were carried out using the PyTorch library and Python 3.7 where PyTorch was used for the feature extraction part using a deep neural network and machine learning algorithms were used from the Scikit-learn library. We did all investigation on an Intel® Xeon® CPU E5-2697 v4 @ 2.30 GHz with 64 GB RAM and a 16 GB NVIDIA GeForce GTX 1080 GPU.

As stated in the earlier section, two different types of investigations were carried out: risk classification and outcome prediction for high-risk patients. Fivefold cross-validation was performed in this study. Therefore, 80% of the data were used for training and 20% for testing in each fold. Finally, a weighted average of the five folds was calculated. The number of training and test Chest X-ray images, and clinical data used in the two experiments are listed in Table 2.

**Table 2:** Details of the dataset used for training, validation, and testing

Database	Types	# of patients	Training data/fold	Augmented training data/fold	Test data/fold
Risk classification	Low	396	317	317 × 4 = 1268	79
	High	534	427	427 × 3 = 1281	107
Outcome prediction	Survived	364	291	291 × 4 = 1164	73
	Death	170	136	136 × 9 = 1224	34

#### Development and internal validation of stacking classification model

We have used reduced features after using PCA from CXR images and top-ranked clinical features individually and in combination and used for risk and death prediction using eight machine learning classifiers, namely Random Forest [55], Support Vector Machine (SVM) [56], K-nearest neighbor (KNN) [57], XGBoost [58], Adaboost [59], Gradient boosting, linear discriminant analysis (LDA) [60], and Logistic regression [61]. This study used fivefold stratified cross-validation where four folds are used to generate a training set for the classifiers and leave onefold for validation. The best-performing three models were used to train, validate, and test the Stacking model (as described earlier).

#### Experiment-01: risk stratification using CXR Image and clinical data

In this experiment, we investigated three different experiments to predict the risk of COVID-19 patients. The first one is conducted on CXR image features, while the second one is carried on Clinical features, and finally, the combined features from both modalities are used to stratify the risk.

A. Binary classification (low vs high risk) using CXR images

The CheXNet model was used to extract features from CXR and then PCA was used to reduce the dimensionality of the CXR features. Then, using reduced feature components and fivefold cross-validation, eight alternative ML classifiers were developed to determine which models performed well in classifying low and high-risk patients. The stacking model was built using the top three base models and a meta-model, and the performance of the stacking technique for the CXR image alone is reported.

B. Binary classification (low vs high risk) using clinical data

Using fivefold cross-validation, Top-5 features (LDH, O_2_ percentage, Age, WBC, and CRP) identified in the previous stage were tested on eight different ML classifiers to determine which models performed best in classifying low and high-risk patients. A stacking model was trained using the top-performing three algorithms as base models to train a meta learner and the performance of the meta learner and base models are reported.

C. Binary classification (low vs high risk) using CXR images and clinical data

The performance of decreased CXR feature components and top-ranked clinical variables in categorizing low- and high-risk patients using different ML classifiers for fivefold cross-validation was crucial to determine. This experiment will demonstrate the efficacy of the multimodal method presented in this work in comparison to the hundreds of approaches published on CXR alone and the tens of approaches published on clinical data alone.

#### Experiment-02: death probability prediction for high-risk patients

We studied three investigations to predict the death outcome of high-risk COVID-19 patients, as shown in Experiment-01. The first one is conducted on CXR image features, while the second one is carried on Clinical features, and finally, the combined features from both modalities are used to stratify the dead and survived patients.

A. Binary classification (survival vs death) using CXR images

The features extracted from the CXR images using ChexNet were dimensionality reduced using PCA and used to train eight different ML classifiers to see which models performed well in predicting the mortality outcome of high-risk patients using fivefold cross-validation. Among the eight models, the best performing three models were used to train the stacking model and the results of base and stacking models are reported.

B. Binary classification (survival vs death) using clinical data

Top-5 clinical features (LDH, O_2_ percentage, Age, WBC, and CRP) were tested on eight different ML classifiers to determine which models performed best in predicting the mortality outcome among high-risk patients. A stacking model was trained using the top-performing three algorithms as a base model to train a meta learner and the performance of the meta learner and base models are reported.

C. Binary classification (survival vs death) using CXR images and clinical data

As a multimodal approach, we have investigated the efficacy of reduced CXR features and top-ranked clinical features to predict the mortality outcome of high-risk patients using fivefold cross-validation using the same eight models. Then the Top-3 best performing models were used to train the Stacking ML model and the results for base models and stacking model were reported.

#### Development and validation of logistic regression-based nomogram

Nomograms are a popular graphical scoring technique for converting statistical models into an estimate of the probability of a single event [70]. This can be accomplished using various ML classifiers, such as the Logistic regression classifier. Multiple independent predictors (x) are utilized by logistic regression to predict linearly related outcomes (y). Using linear prediction, the event probability (Pr) can be computed, and the results can be reported. A logistic regression-based nomogram was developed for patients at high risk to stratify their survival and mortality rates. Using the integrated features from CXR and clinical data as well as the base learners' prediction, logistic regression was used to create a nomogram. In addition, calibration curves for model development and validation were plotted to compare the projected and actual death probability of high-risk patients. In addition, decision curve analysis was utilized to finalize the threshold probability ranges within the clinically useful range of the nomograms.

### Performance metrics

Recall/Sensitivity (*R*), Precision (*P*), Accuracy (*A*), Specificity (*S*), and *F*1-Score (*F*1) were used to evaluate the performance of different classifiers in the literatures [59]. The results of this study were drawn from the full dataset because fivefold cross-validation was used (five test fold-concatenated). Since the number of occurrences in each class varies, we gave weighted values for both classes and total accuracy. Area under the curve (AUC) was used in judging the model performance. Equations (3–7) depict the mathematical expressions of five evaluation metrics:

3 $$ A = \frac{{{\text{TP}}_{{{\text{class}}\_i}} + {\text{TN}}_{{{\text{class}}\_i}} }}{{{\text{TP}}_{{{\text{class}}\_i}} + {\text{TN}}_{{{\text{class}}\_i}} + {\text{FP}}_{{{\text{class}}\_i}} + {\text{FN}}_{{{\text{class}}\_i}} }} $$

4 $$ P = \frac{{{\text{TP}}_{{{\text{class}}\_i}} }}{{{\text{TP}}_{{{\text{class}}\_i}} + {\text{FP}}_{{{\text{class}}\_i}} }} $$

5 $$ R = \frac{{{\text{TP}}_{{{\text{class}}_{i} }} }}{{{\text{TP}}_{{{\text{class}}_{i} }} + {\text{FN}}_{{{\text{class}}_{i} }} }} $$

6 $$ F1 = 2\frac{{{\text{Precision}}_{{{\text{class}}_{i} }} \times {\text{Sensitivity}}_{{{\text{class}}_{i} }} }}{{{\text{Precision}}_{{{\text{class}}_{i} }} + {\text{Sensitivity}}_{{{\text{class}}_{i} }} }} $$

7 $$ S = \frac{{{\text{TN}}_{{{\text{class}}\_i}} }}{{{\text{TN}}_{{{\text{class}}\_i}} + {\text{FP}}_{{{\text{class}}\_i}} }} $$

$$ {\text{where}}\;{\text{class}}_{i} = {\text{Mild}}\;{\text{and}}\;{\text{severe}}\;{\text{or}}\;{\text{survived}}\;{\text{and}}\;{\text{death}} $$

where ${\text{TP}}_{{{\text{class}}\_i}}$ is true positive, indicating correct detection of the actual class, ${\text{TN}}_{{{\text{class}}_{i} }}$ is true negative, indicating correct detection of the other classes, ${\text{FP}}_{{{\text{class}}\_i}}$ is false positive, indicating incorrect detection of the other classes, and ${\text{FN}}_{{{\text{class}}\_i}}$ is false negative, indating incorrect detection of the actual class.

## Results

### Best features and their combination selection

**Fig. 6:**
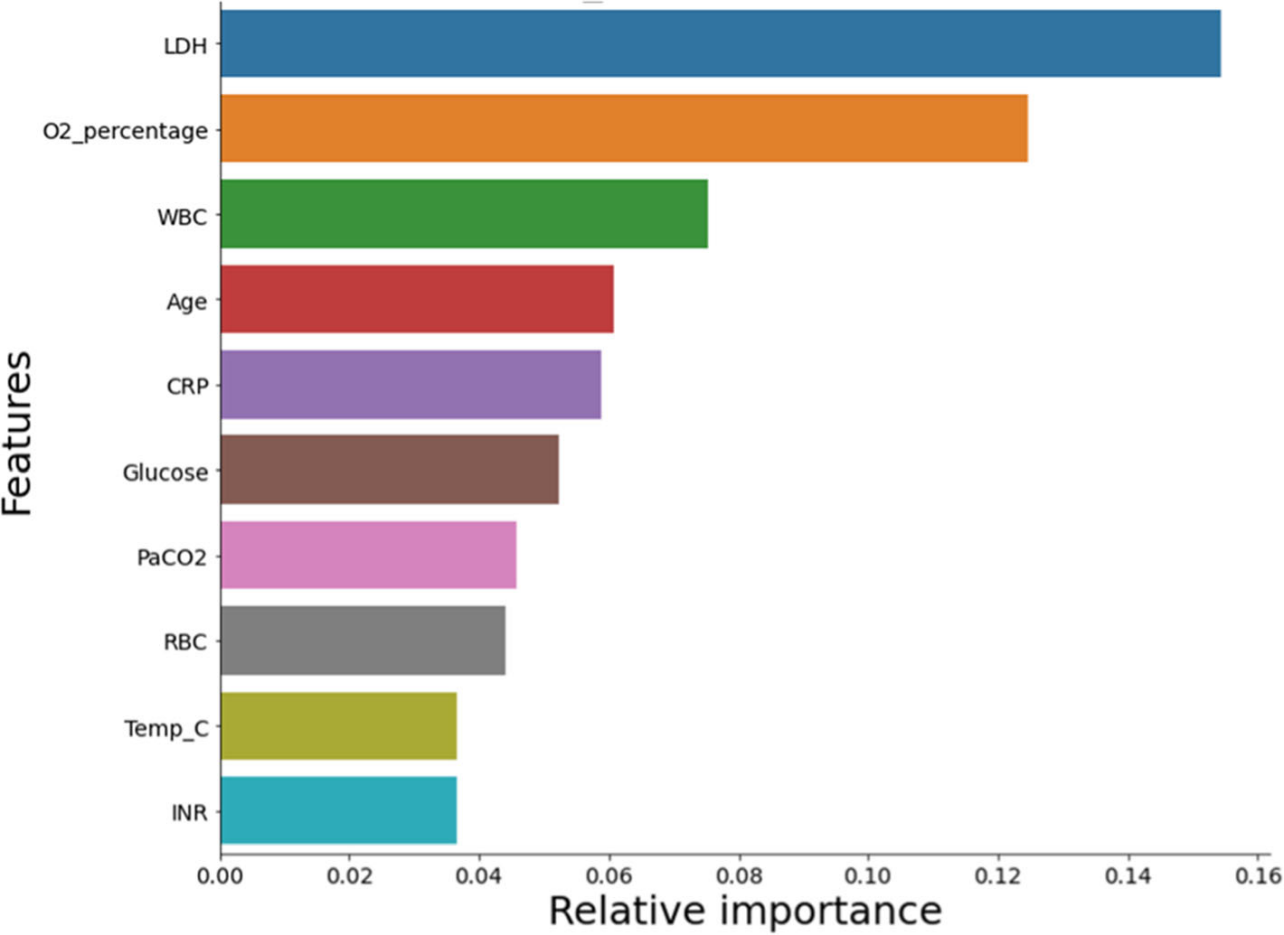
Top ten features selected using the random forest feature selection technique

The random forest feature ranking technique was used to select the top-ranked ten features from 25 statistically significant features (Fig. 6). Moreover, we used few fine-tuned parameters for Random Forest features selection technique using Optuna optimizer [71] and we trained the algorithm with ‘n_estimators’ = 75, ‘criterion’ = ‘entrophy’, and ‘max_depth’ = 50. Table 3 shows the results of testing these top-ranked 10 features with multiple classifiers to determine the best-performing feature combinations. When using the top-ranked 5 features, the Gradient Boosting classifier outperforms other networks in binary classification (low- vs. high-risk). Gradient Boosting produces overall accuracy, weighted sensitivity, precision, specificity, and F1 scores of 82.91%, 82.91%, 82.87%, 82.91%, and 82.87%, respectively, when only the Top-5 characteristics are used (LDH, O_2_ percentage, WBC, Age, and CRP). Among the Top-10 features, determining the most appropriate parameters for the early prediction of high-risk COVID-19 patients was critical.

**Table 3:** Summary of the performance metrics for Top 1 to 10 clinical features

Feature combination	Weighted average (95% confidence interval)				
	*P*	*R*	*F*1	*S*	*A*
Top 1 feature	59.62 ± 6.33	59.71 ± 6.33	59.62 ± 6.33	59.62 ± 6.33	59.64 ± 6.33
Top 2 features	60.26 ± 6.31	60.26 ± 6.31	60.26 ± 6.31	60.26 ± 6.31	60.26 ± 6.31
Top 3 features	80.77 ± 5.08	80.8 ± 5.08	80.77 ± 5.08	80.77 ± 5.08	80.73 ± 5.09
Top 4 features	78.21 ± 5.32	78.22 ± 5.32	78.21 ± 5.32	78.21 ± 5.32	78.16 ± 5.33
Top 5 features	82.91 ± 1.83	82.87 ± 1.83	82.91 ± 1.83	82.91 ± 1.83	82.74 ± 1.84
Top 6 features	79.49 ± 5.21	79.58 ± 5.2	79.49 ± 5.21	79.49 ± 5.21	79.42 ± 5.21
Top 7 features	76.28 ± 5.49	76.39 ± 5.48	76.28 ± 5.49	76.28 ± 5.49	76.18 ± 5.49
Top 8 features	76.92 ± 5.43	77.22 ± 5.41	76.92 ± 5.43	76.92 ± 5.43	76.75 ± 5.45
Top 9 features	75 ± 5.58	75.33 ± 5.56	75.00 ± 5.58	75.00 ± 5.58	74.78 ± 5.6
Top 10 features	76.28 ± 5.49	76.39 ± 5.48	76.28 ± 5.49	76.28 ± 5.49	76.28 ± 5.49

### Risk prediction of COVID-19 patients

In this section, the results of three different experiments to predict low or high-risk COVID-19 patients were reported. The performance of different ML models for CXR images, then using clinical data was reported separately and in combination. Each of these results is based on fivefold cross-validation.

#### Performance analysis using CXR images

The gradient boosting classifier was the best performing classifier for stratifying the low- and high-risk COVID-19 patients. It achieves precision, sensitivity, and F1 scores of 78.41%, 78.48%, and 78.41%, respectively. The stacking model was built using the top three classifiers such as Random Forest, KNN, and Gradient Boosting. The stacking model produces slightly better performance with precision, sensitivity, and F1 scores of 79.5%, 79.53%, and 79.54%, respectively.

#### Performance analysis using clinical data

The gradient boosting classifier outperforms other classifiers in binary classification with precision, sensitivity, and F1 scores of 82.81%, 82.8%, and 82.81%, respectively. The stacking model was trained using the top three algorithms (Random Forest, Gradient Boosting, and XGBoost). A meta-learner logistic regression classifier was used and outperformed the base model with precision, sensitivity, and F1 scores of 83.01%, 83.87%, and 83.01%, respectively.

#### Performance analysis using both CXR images and clinical data

The gradient boosting classifier outperforms other classifiers with precision, sensitivity, and F1 scores of 88.81%, 88.81%, and 88.81%, respectively, using combined CXR characteristics and clinical data. The stacking model was built using the top three algorithms (Gradient Boosting, LDA, and Random Forest) and it outperforms the base models and produces precision, sensitivity, and F1 scores of 89.03%, 90.44%, and 89.03%, respectively. Using a combination of CXR and top-ranked clinical characteristics, the stacking model revealed around a 6% improvement. Table 4 compares, with a 95% confidence interval, the prediction of low- or high-risk patients using CXR characteristics and clinical data alone and in combination with different classifiers employing distinct metrics.

**Table 4:** Comparison of performance metrics for risk prediction using different ML models and approaches (single mode and multimode)

Dataset	Classifier	Overall	Weighted with 95% CI			
		*A*	*P*	*R*	*F*1	*S*
CXR images	Linear discriminant analysis (LDA)	71.75 ± 2.97	71.75 ± 2.97	71.75 ± 2.97	71.75 ± 2.97	71.54 ± 3
	XGBoost (XGB)	73.69 ± 2.62	73.7 ± 2.62	73.69 ± 2.62	73.69 ± 2.62	73.68 ± 2.63
	Random forest (RF)	76.26 ± 3.05	76.3 ± 3.04	76.26 ± 3.05	76.25 ± 3.05	75.79 ± 3.12
	Logistic regression (LR)	71.18 ± 2.36	71.63 ± 2.4	71.24 ± 2.19	71.92 ± 2.3	71.31 ± 2.57
	Support vector machine (SVM)	74.26 ± 3.05	74.26 ± 3.05	74.26 ± 3.05	74.26 ± 3.05	74.96 ± 3.09
	Extra tree (ET)	70.29 ± 3.19	70.32 ± 3.19	70.29 ± 3.19	70.3 ± 3.19	70.32 ± 3.19
	K-nearest neighbors (KNN)	75.66 ± 2.43	75.66 ± 2.43	75.66 ± 2.43	75.66 ± 2.43	74.5 ± 2.46
	Gradient boosting (GB)	78.41 ± 3.26	78.48 ± 3.27	78.41 ± 3.26	78.44 ± 3.27	77.9 ± 3.3
	Stacking model (RF + KNN + GB)	79.5 ± 1.97	79.53 ± 1.98	79.54 ± 1.97	79.54 ± 1.97	79.45 ± 1.98
Clinical data	Linear discriminant analysis (LDA)	78.75 ± 2.89	78.75 ± 2.89	78.75 ± 2.89	78.75 ± 2.89	78.54 ± 2.9
	XGBoost (XGB)	80.69 ± 2.79	80.7 ± 2.79	80.69 ± 2.79	80.69 ± 2.79	80.68 ± 2.79
	Random forest (RF)	81.66 ± 2.73	81.66 ± 2.73	81.66 ± 2.73	81.66 ± 2.73	81.5 ± 2.74
	Logistic regression (LR)	74.81 ± 2.72	74.8 ± 2.73	74.81 ± 2.72	74.8 ± 2.73	74.56 ± 2.74
	Support vector machine (SVM)	78.26 ± 2.91	78.26 ± 2.91	78.26 ± 2.91	78.26 ± 2.91	77.96 ± 2.93
	Extra tree (ET)	77.29 ± 2.96	77.32 ± 2.96	77.29 ± 2.96	77.3 ± 2.96	77.32 ± 2.96
	K-nearest neighbors (KNN)	76.26 ± 2.64	76.26 ± 2.64	76.26 ± 2.64	76.26 ± 2.64	76.96 ± 2.66
	Gradient boosting (GB)	82.91 ± 1.83	82.87 ± 1.83	82.91 ± 1.83	82.91 ± 1.83	82.74 ± 1.84
	Stacking model (GB + RF + XGB)	83.01 ± 3.15	83.87 ± 3.14	83.01 ± 3.14	83.01 ± 3.14	83.04 ± 3.17
Both CXR images and clinical data	Linear discriminant analysis (LDA)	83.26 ± 2.51	83.26 ± 2.51	83.26 ± 2.51	83.26 ± 2.51	82.96 ± 2.53
	XGBoost (XGB)	85.69 ± 2.35	85.7 ± 2.35	85.69 ± 2.35	85.69 ± 2.35	85.68 ± 2.35
	Random forest (RF)	83.75 ± 2.48	83.75 ± 2.48	83.75 ± 2.48	83.75 ± 2.48	83.54 ± 2.49
	Logistic regression (LR)	82.29 ± 2.57	82.39 ± 2.56	82.29 ± 2.57	82.31 ± 2.57	82.49 ± 2.55
	Support vector machine (SVM)	81.66 ± 2.4	81.66 ± 2.4	81.66 ± 2.4	81.66 ± 2.4	81.5 ± 2.41
	AdaBoost	80.75 ± 2.61	80.75 ± 2.61	80.75 ± 2.61	80.75 ± 2.61	80.54 ± 2.62
	K-nearest neighbors (KNN)	76.66 ± 2.29	76.66 ± 2.29	76.66 ± 2.29	76.66 ± 2.29	76.5 ± 2.3
	Gradient boosting (GB)	88.81 ± 2.59	88.8 ± 2.59	88.81 ± 2.59	88.8 ± 2.59	88.56 ± 2.61
	Stacking model (GB + XGB + RF)	89.03 ± 2.18	90.44 ± 2.15	89.03 ± 2.15	89.03 ± 2.15	88.7 ± 2.2

**Fig. 7:**
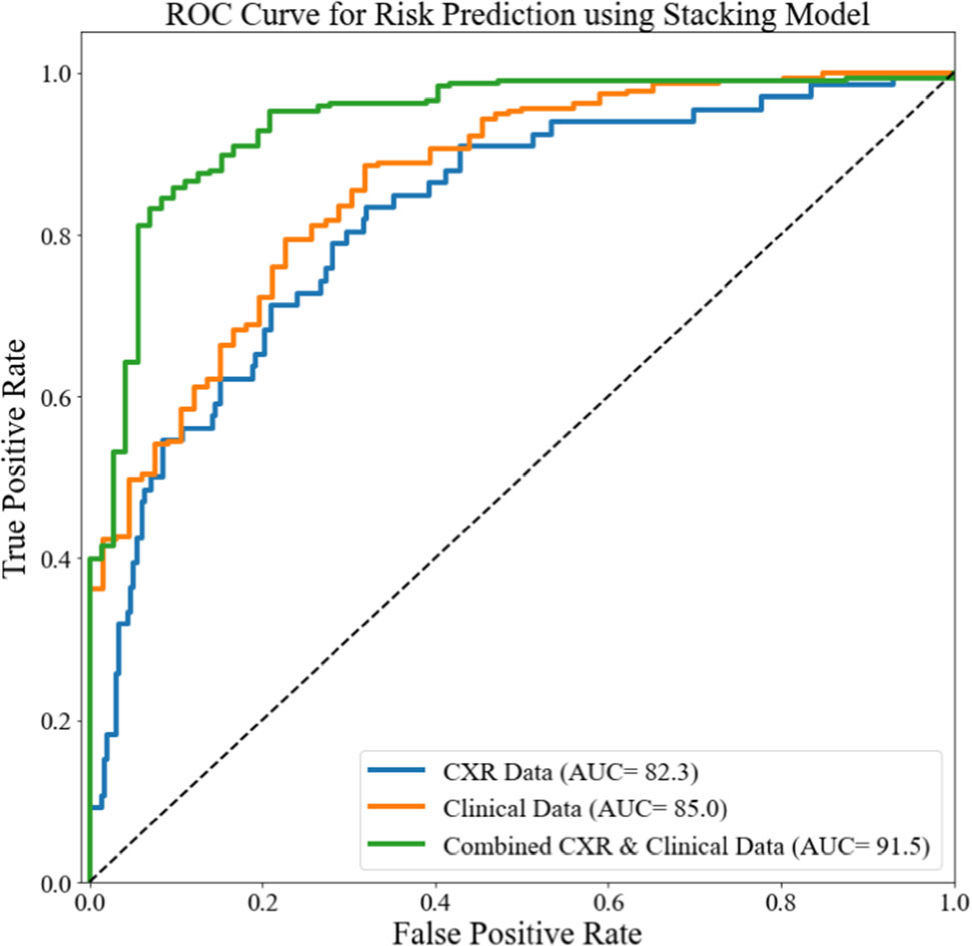
ROC curves for risk prediction of COVID-19 patients with single and multi-modal data using the stacking ML model

In Fig. 7, it can be seen that combined CXR image features and clinical top-ranked features outperformed individual modality with an AUC of 91.5%. The AUC values for CXR image features and clinical top-ranked features individually using the stacking model produced 82.3% and 85% of AUC, respectively.

### Death probability prediction for high-risk patients

In this section, the results of three different experiments to predict the probability of death among high-risk COVID-19 patients were reported. The fivefold performance of different ML models for CXR images, then using clinical data were reported separately.

#### Performance analysis with CXR images

Random Forest classifier outperforms the other 7 classifiers in classifying the dead and survived COVID-19 patients with precision, sensitivity, and F1 scores of 84.83%, 85.02%, and 84.83%, respectively. The stacking model was built using the top three methods (Random Forest, Extra Tree, and Gradient Boosting) and produces precision, sensitivity, and F1 scores of 86.35%, 83.22%, and 86.35%, respectively.

#### Performance analysis with clinical data

The gradient boosting model outperforms the other seven classifiers in stratifying the survival and dead patients with precision, sensitivity, and F1 scores of 89.14%, 89.86%, and 89.14%, respectively. The stacking model was trained using the top three models (Random Forest, XGBoost, and Extra Tree). The stacking model beat previous base models, achieving 91.2% precision, 91.25% sensitivity, and 91.2% F1 scores, respectively.

#### Performance analysis using both CXR images and clinical data

Random Forest classifier outperforms other models with precision, sensitivity, and F1 scores of 91.76%, 91.86%, and 91.76%, respectively. The stacking machine learning model was trained using Random Forest, Extra Tree, and Gradient Boosting and it outperforms the base model with precision, sensitivity, and F1 scores of 92.88%, 93.37%, and 92.88%, respectively. In terms of all the different performance metrics, the performance of the stacking model improved by ~ 6% when using both reduced CXR features and clinical top features, refer to Table 5. Moreover, the finetuned parameters for the best performing classifiers are shown in Supplementary Table 1.

**Table 5:** Comparison of performance metrics for death prediction using different ML models and approaches (single mode and multimode)

Dataset	Classifier	Overall	Weighted with 95% CI			
		*A*	*P*	*R*	*F*1	*S*
CXR images	Linear discriminant analysis (LDA)	68.35 ± 4.41	73.22 ± 4.2	68.35 ± 4.41	68.35 ± 4.41	69.4 ± 4.37
	XGBoost (XGB)	73.03 ± 4.21	75.9 ± 4.06	73.03 ± 4.21	73.03 ± 4.21	73.79 ± 4.17
	Random forest (RF)	84.83 ± 3.4	85.02 ± 3.38	84.83 ± 3.4	84.83 ± 3.4	84.91 ± 3.4
	Logistic regression (LR)	67.6 ± 4.44	72.65 ± 4.23	67.6 ± 4.44	67.6 ± 4.44	68.69 ± 4.4
	Support vector machine (SVM)	54.87 ± 4.72	59.08 ± 4.66	54.87 ± 4.72	54.87 ± 4.72	56.26 ± 4.71
	Extra tree (ET)	82.4 ± 3.61	82.72 ± 3.59	82.4 ± 3.61	82.4 ± 3.61	82.53 ± 3.6
	K-nearest neighbors (KNN)	72.85 ± 4.22	76.51 ± 4.02	72.85 ± 4.22	72.85 ± 4.22	73.68 ± 4.18
	Gradient boosting (GB)	80.71 ± 3.74	82.59 ± 3.6	80.71 ± 3.74	80.71 ± 3.74	81.17 ± 3.71
	Stacking model (RF + ET + GB)	86.35 ± 3.26	83.22 ± 3.54	86.35 ± 3.26	86.35 ± 3.26	87.4 ± 3.15
Clinical data	Linear discriminant analysis (LDA)	69.29 ± 4.38	73.74 ± 4.17	69.29 ± 4.38	69.29 ± 4.38	70.29 ± 4.33
	XGBoost (XGB)	87.08 ± 3.18	87.73 ± 3.11	87.08 ± 3.18	87.08 ± 3.18	87.26 ± 3.16
	Random forest (RF)	89.14 ± 2.95	89.86 ± 2.86	89.14 ± 2.95	89.14 ± 2.95	89.31 ± 2.93
	Logistic regression (LR)	68.91 ± 4.39	73.37 ± 4.19	68.91 ± 4.39	68.91 ± 4.39	69.92 ± 4.35
	Support vector machine (SVM)	53.56 ± 4.73	58.01 ± 4.68	53.56 ± 4.73	53.56 ± 4.73	55.02 ± 4.72
	Extra tree (ET)	86.33 ± 3.26	86.31 ± 3.26	86.33 ± 3.26	86.33 ± 3.26	86.32 ± 3.26
	K-nearest neighbors (KNN)	75.84 ± 4.06	79.56 ± 3.82	75.84 ± 4.06	75.84 ± 4.06	76.6 ± 4.02
	Gradient boosting (GB)	71.72 ± 4.27	71.42 ± 4.29	71.72 ± 4.27	71.72 ± 4.27	71.56 ± 4.28
	Stacking model (RF + XGB + ET)	91.2 ± 2.69	91.25 ± 2.68	91.2 ± 2.69	91.2 ± 2.69	91.22 ± 2.68
Both CXR images and clinical data	Linear discriminant analysis (LDA)	74.34 ± 4.14	78.91 ± 3.87	74.34 ± 4.14	74.34 ± 4.14	75.19 ± 4.1
	XGBoost (XGB)	77.53 ± 3.96	80.24 ± 3.78	77.53 ± 3.96	77.53 ± 3.96	78.15 ± 3.92
	Random forest (RF)	91.76 ± 2.61	91.86 ± 2.59	91.76 ± 2.61	91.76 ± 2.61	91.8 ± 2.6
	Logistic regression (LR)	79.78 ± 3.81	85.84 ± 3.31	79.78 ± 3.81	79.78 ± 3.81	80.47 ± 3.76
	Support vector machine (SVM)	69.29 ± 4.38	75.98 ± 4.05	69.29 ± 4.38	69.29 ± 4.38	70.34 ± 4.33
	Extra tree (ET)	90.07 ± 2.84	90.65 ± 2.76	90.07 ± 2.84	90.07 ± 2.84	90.22 ± 2.82
	K-nearest neighbors (KNN)	81.84 ± 3.66	83.8 ± 3.49	81.84 ± 3.66	81.84 ± 3.66	82.28 ± 3.62
	Gradient boosting (GB)	88.95 ± 2.97	90.21 ± 2.82	88.95 ± 2.97	88.95 ± 2.97	89.19 ± 2.95
	Stacking model (RF + ET + GB)	92.88 ± 2.44	93.37 ± 2.36	92.88 ± 2.44	92.88 ± 2.44	92.65 ± 2.48

**Fig. 8:**
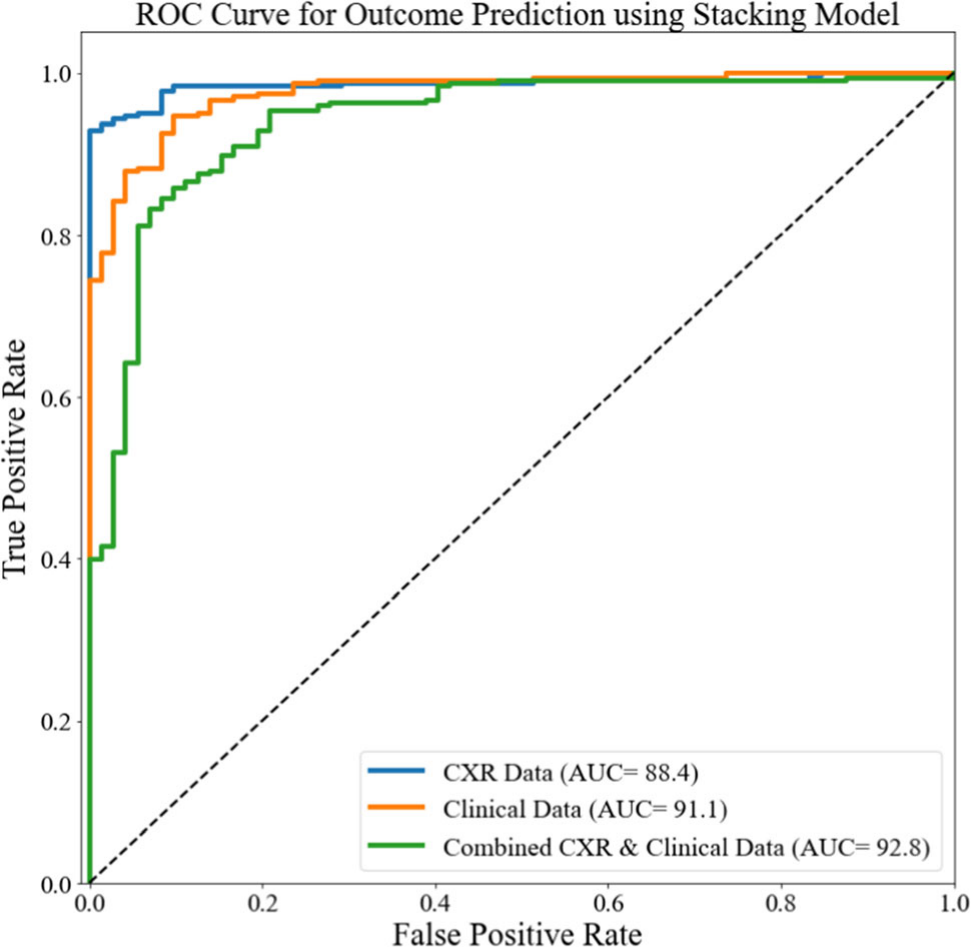
ROC curves for outcome prediction of high-risk patients with single and multi-modal data using the stacking ML model

In Fig. 8, it also can be visible that combined CXR image features and clinical top-ranked features outperformed individual modalities with an AUC of 92.8%. The reduced CXR image features and clinical top-ranked features using the stacking model individually produce an AUC of 88.4% and 91.1%, respectively. In this study, the main contributing parameter which helped to improve the result was the PCA variance. PCA was used to reduce the dimensionality of the extracted features from the images using CNN encoder with different variance from 70 to 95% to produce the best performance. The performance for single and multimodal data with different PCA variance using stacking model are shown in Supplementary Tables 2 and 3 for study 1 and 3, respectively.

#### Stacking ML-based nomogram

Due to the superior performance of the Logistic regression meta-learner in the classification of survival and death patients, a Nomogram was created using the probability scores of the three best models (Random Forest (M1), Extra Tree (M2), and Gradient Boosting (M3)) to accurately estimate the survival and death probabilities of the high-risk group. Using multivariate logistic regression, the relationship between the probability scores of these base learner models and the likelihood of death in high-risk patients was explored (Table 6). Using the z-value, which is determined using the regression coefficient and standard error, is a common way of detecting relevant characteristics. High z-values indicate that the independent variable is significant.

**Table 6:** Summary of logistic regression analysis

Outcome	Coefficient	Bootstrap std. error	*Z*	*P* >|*z*|	[95% CI]	
Random forest	− 14.85299	3.262317	− 4.5	0.000	− 21.24701	− 8.458965
Extra tree	− 5.028269	5.40965	− 0.93	0.353	− 15.63099	5.57445
Gradient boosting	− 1.788734	0.47932	− 3.73	0.000	− 2.728183	− 0.84928
Cons	11.23907	1.822279	6.17	0.0004	7.667468	14.81067

Table 6 demonstrates that Extra Tree (*M*2) is not a particularly accurate predictor of COVID-19 individuals, although Random Forest (*M*1) and Gradient Boosting (*M*3) are accurate predictors. If *p* < 0.05, the *p* value can be utilized to identify a significant variable; *X*-variables may have a substantial relationship with *Y*-variables. The *p* value also demonstrates that the Extra Tree model is a weak predictor. The linear prediction (LP) and Probability of death in high-risk patients (Prob) are calculated using Eqs. 8–9.

Nonetheless, it was noticed that the model performance was marginally diminished when two models were stacked instead of three. Therefore, three models are utilized to produce the Nomogram. As seen in Fig. 8, the nomogram comprises six rows, running from 1 to 3, to represent the variables included. The “Points axis” produced a score for each variable in the high-risk death or survival category. The total score was displayed in row 6 after being determined by adding the points from the three factors (row 4). To determine a patient's mortality risk, a line is drawn from the “Total Score” axis to the “Prob” axis (row 5).

Alternatively, the following formula can be used to calculate the nomogram score:

8 $$ {\text{LP}} = 11.23907 - 14.85299 \times M1 - 5.028269 \times M2 - 1.788734 \times M3 $$

9 $$ {\text{Prob}} = 1/ \left( {1 + \exp \left( { - {\text{LP}}} \right)} \right) $$

**Fig. 9:**
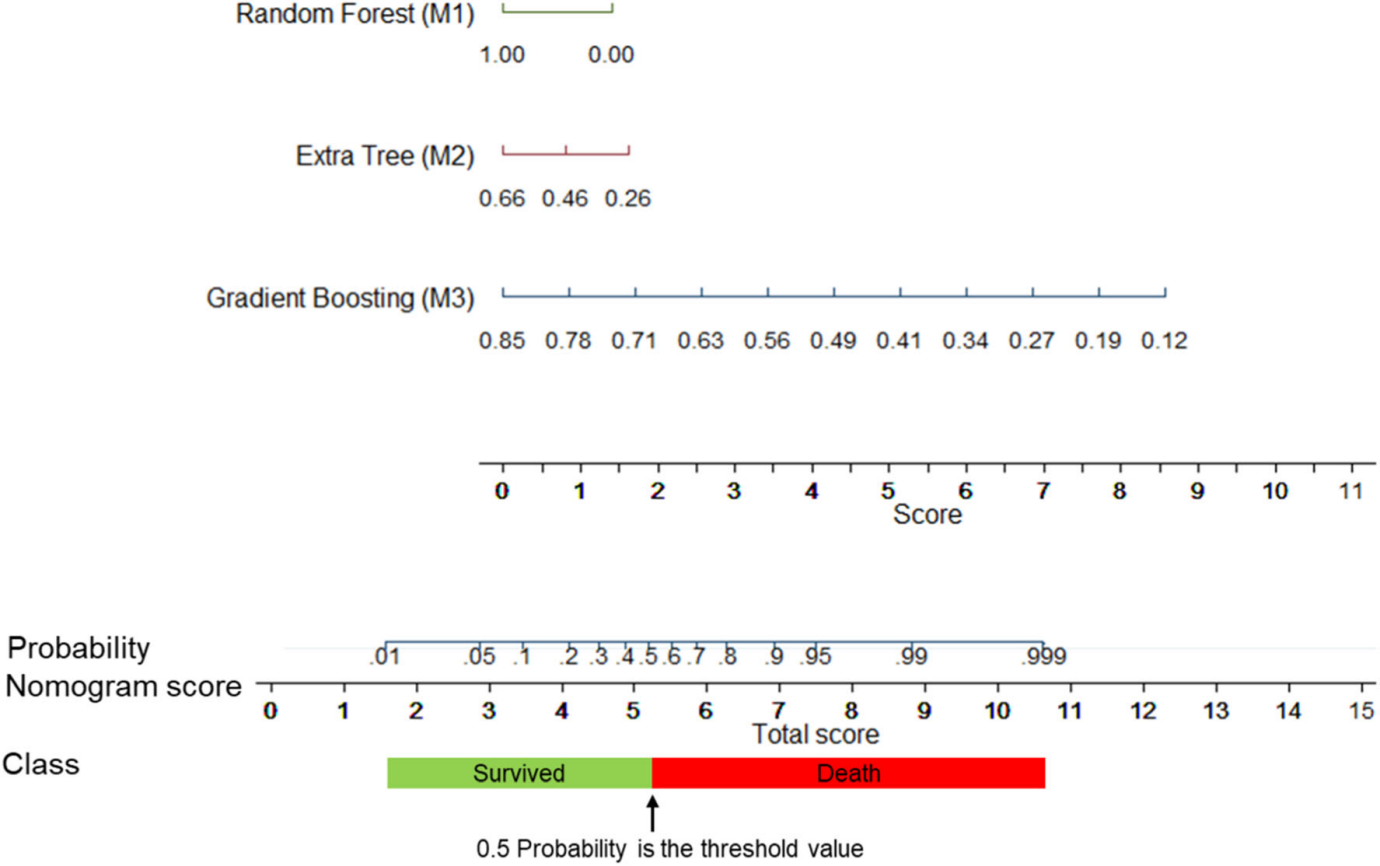
A Nomogram for prediction of death in COVID-19 severe patients was created using Random Forest ($M1$), ExtraTree ($M2$), and Gradient boosting ($M3$)

Figure 9 also depicts the Nomogram scores for both the survived and the deceased classes. It was discovered that 50% classification probability cutoffs correspond to a Nomogram score of 4.8 or a probability of 0.5, stratifying the classes.

**Fig. 10:**
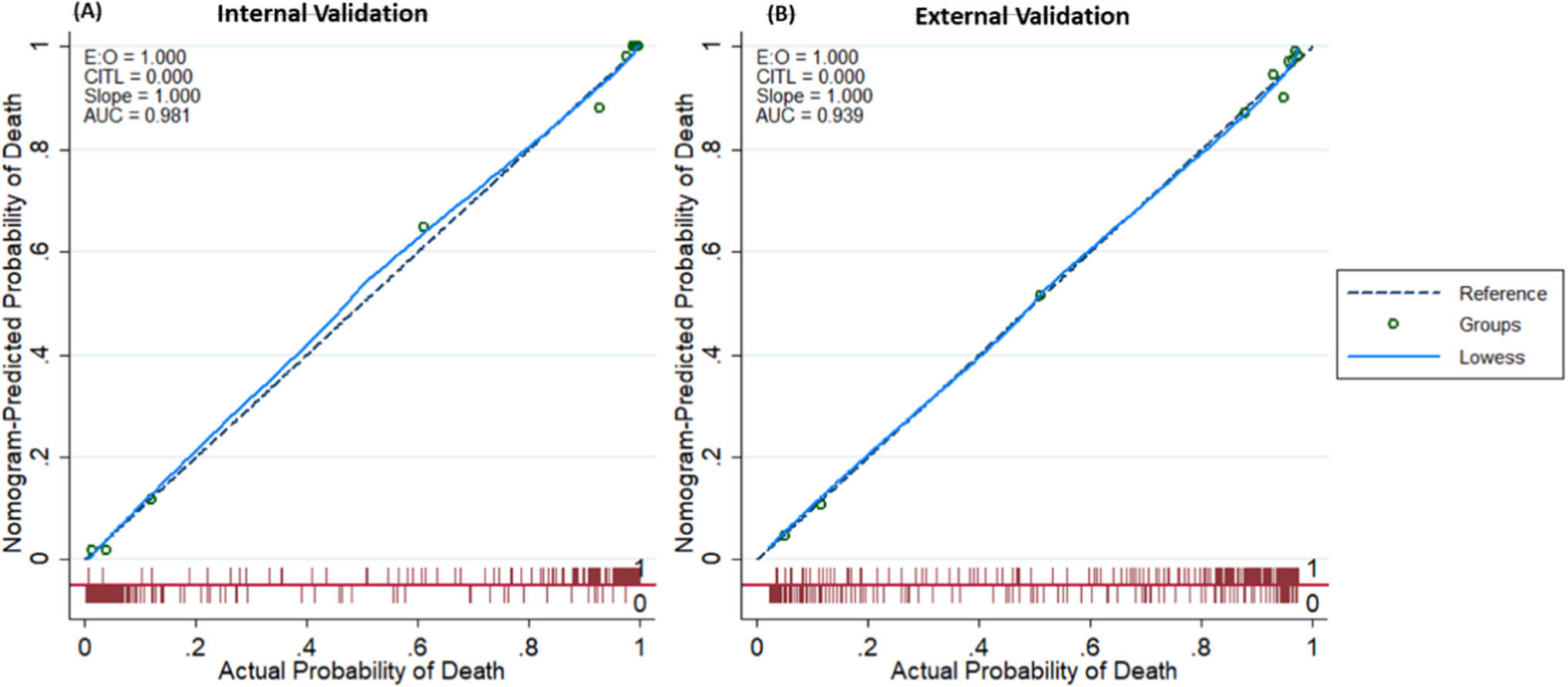
**A** Internal validation calibration plot, **B** External validation calibration plot

Figure 10 depicts both the internal and external validation calibration plots. It demonstrates that each calibration curve is extremely near to the diagonal line, indicating a valid model. The AUC values for internal and external validation are 98.1% and 93.8%, respectively, which demonstrates the model’s exceptional performance.

**Fig. 11:**
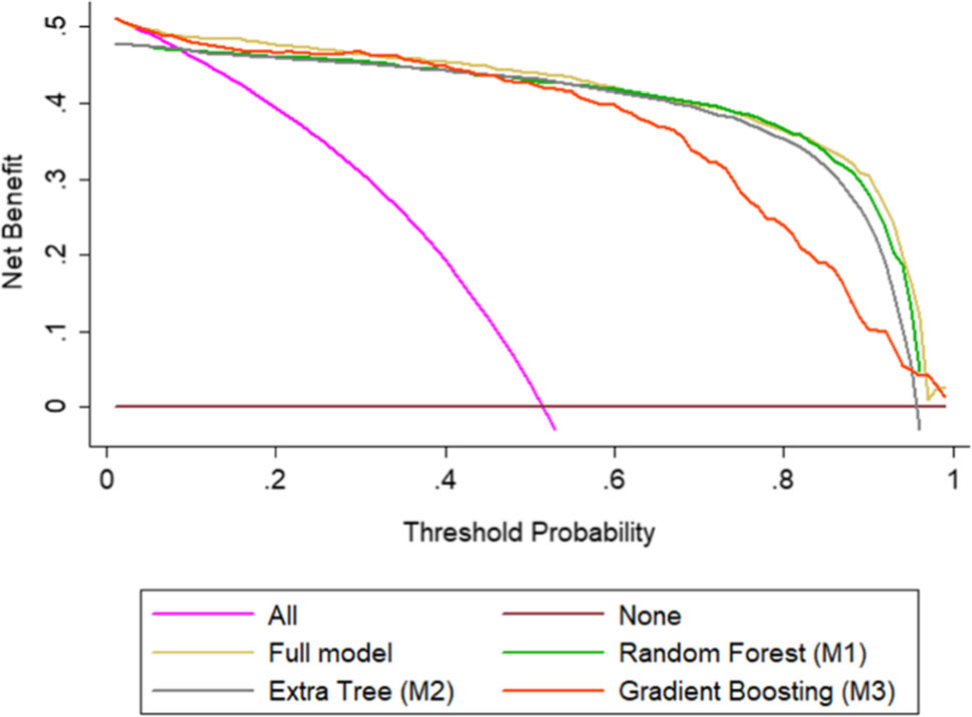
Decision curves analysis comparing different models to predict the death probability of patients with high-risk COVID-19

Figure 11 illustrates that the net benefit of each predictor model was positive (threshold 0.95), showing that each predictor contributed to the prediction of the outcome. Particularly, the whole model produced the most accurate results, necessitating the employment of three base models as predictors in the Stacking model.

#### Performance evaluation of the model

Using the Nomogram score, we compared the actual death rate to the projected death rate among high-risk people. Table 7(A) demonstrates that the proportions of death outcomes in the training set were 91.9% (125/136) for the death group and 8.1% (11/136) for the surviving group, while the proportions of death outcomes in the test set were 91.18% (31/34) for the death group and 8.82% (3/34) for the survived group (Table 7(B)). The actual mortality rates varied considerably between the two groups (p 0.001). Consequently, this scoring method can be utilized to predict patient outcomes.

**Table 7:** Performance evaluation of the model in the training cohort (A) and testing cohort (B) using Fisher’s exact probability test

Prediction	Outcome	
	Survived	Death
(A)		
Survived	280 (96.22%)	11 (8.1%)
Death	11 (3.78%)	125 (91.9%)
Overall	291 (100%)	136 (100%)
(B)		
Survived	68 (93.1%)	3 (8.82%)
Death	5 (6.9%)	31 (91.18%)
Overall	73 (100%)	34 (100%)

#### Web application with back-end server

As an extension of this work, we developed an online application (https://qu-mlg.com/projects/covid-severity-grading-AI) that allows clinicians to input demographic and clinical data (LDH, O_2_ percentage, WBC, age, and CRP) as well as CXR images. BIO-CXRNET is a Google Cloud-based AI application that analyzes data to determine whether a user is a low-risk or high-risk patient. Our model identifies the patient's death risk probability if the patient is in the extreme risk group.

The backend application is written in Python using the Flask framework. Python’s Flask is a strong backend application framework. The cloud application is deployed on an Apache 2.0 HTTP server using Ubuntu 20.01 LTS Google Computation Engine (GCE). To reduce server costs, a GCE instance with minimal configuration is hired. The GCE server is equipped with a 4-core Intel Xenon processor, 8 GB of DDR4 memory, and 100 GB of balanced persistent storage. To handle the computation-intensive ML models in a resource-constrained context, the operating system kernel configurations are adjusted. Such setups include activating non-threaded pre-forking for the Apache web server so that Tensorflow processes have access to more RAM. This online application was developed with Flutter, a programming language based on Google’s Dart.

**Fig. 12:**
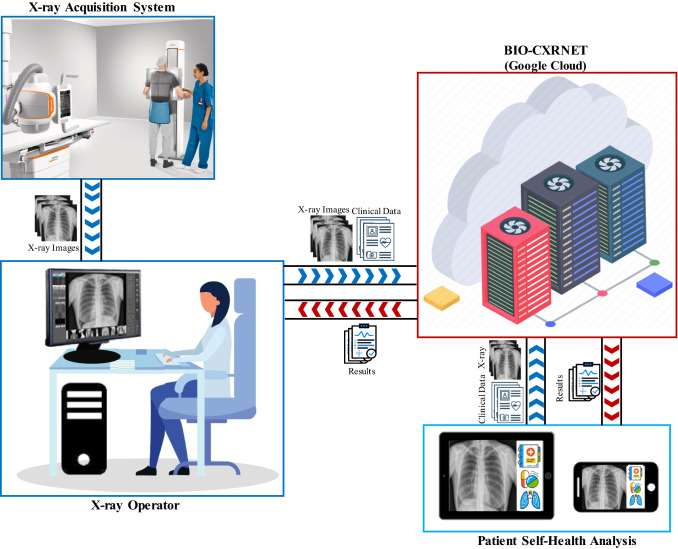
COVID-19 severity risk detection tool using web application framework

In the prototype system, screenshots of the system can be seen in Supplementary Fig. 1, radiologists/clinicians/users will submit demographic information before being prompted to upload CXR image file and four biomarkers, including Lactate Dehydrogenase (LDH) (U/L), Oxygen Saturation (%), White Blood Count (WBC) (10^9L), and C-Reactive Protein (CRP) (mg/dL). CXR image file means the user will give a Chest X-ray image as an input in .png or .jpg format. This will be uploaded to the server, where it will be pre-processed and applied to the BIO-CXRNET model to assess whether the user is a patient at low or high risk (Fig. 12). The data will be processed by the AI backend, and the screen will display a response. The application will display and store the results in a local SQLite database. In conclusion, the application can reduce the load on the healthcare system by expeditiously analyzing the severity risk of COVID patients using a minimum number of blood signs.

## Discussion

This study proposes a multimodal system for forecasting the risk of COVID-19-positive individuals and, as a result, stratifying the probable outcome of patients at high risk. Using CXR images and clinical data, the performances of both of the tests were examined separately and in conjunction. Both experiments demonstrated that the multimodal strategy outperformed the single modality. CXR and clinical features combined demonstrated an accuracy of 89.03% for risk group stratification among COVID-19 patients, compared to 80.11% and 86.01% for CXR and clinical features, respectively. Moreover, in the instance of outcome prediction for high-risk patients, the multimodal technique exceeded individual modality with a 92.3% accuracy, whereas CXR pictures and clinical data alone achieved an accuracy of 89.5% and 90.11%, respectively. As indicated in Table 8, the performance of the results provided in this work is superior to several state-of-the-art performances published in the literature.

**Table 8:** Comparison with state-of-the-art works in the literature

Paper	Method	Dataset	Results
Yan et al. [72]	Predict individual patient mortality using the XGBoost classifier	Clinical biomarkers (485 COVID-19 patients)	Accuracy of 90%
Rahman et al. [65]	Predict COVID-19 severity using machine learning classifiers	Clinical biomarkers (375 COVID-19 patients)	Accuracy of 90.8%
Abbas et al. [73]	CNN (DeTraC)	Chest X-ray images (749 COVID-19)	Accuracy of 93%
Zulfaezal et al. [74]	CNN	Chest X-ray images (1565 COVID-19)	Accuracy of 71.9%
Soda et al. [60]	Deep multimodal CNN	Chest X-ray images and clinical biomarkers (820 COVID-19)	Accuracy of 76.8%
Proposed study	CNN and ML classifiers to predict the severity, and developed nomogram scoring tool	Chest X-ray images and clinical biomarkers (930 COVID-19)	Accuracy of 92.88%

In our previous studies [75] on severe acute respiratory syndrome (SARS) [76], the Middle East respiratory syndrome (MERS) [77], and COVID-19 [78], we discovered that greater age predicted poor outcomes in COVID-19 patients. Since LDH signals tissue/cell death, it is a common indicator of tissue/cell damage. Serum LDH has been recognized as a critical biomarker for the activity and severity of idiopathic pulmonary fibrosis. According to Yan et al. [72], the increase in LDH is one of the most significant prognostic markers of lung injury in patients with the severe pulmonary interstitial illness. The increase in LDH levels in seriously ill COVID-19 patients suggests a worsening of lung injury.

According to studies conducted by Lu et al. [79], CRP testing upon admission is connected with the prediction of short-term mortality related to COVID-19-related diseases. Hepatocytes manufacture CRP when stimulated by cytokines originating from active leukocytes, such as those produced by infections, inflammations, or tissue injury. Hepatocytes manufacture CRP when stimulated by cytokines originating from active leukocytes, such as those produced by infections, inflammations, or tissue injury. Our study indicated that elevated CRP levels upon admission were related to an increased risk of mortality among COVID-19 participants. These data indicated that these patients had developed a significant inflammation or maybe a secondary infection, and antibiotic treatment may be necessary. Increased CRP, a significant indicator of poor prognosis in acute respiratory distress syndrome, suggests a chronic inflammatory state [80, 81]. As a result of this continuous inflammatory response, COVID-19 individuals develop massive gray-white lesions there [82].

Based on prior research, the five biomarkers found in our study were connected with inflammation, immunology, and coagulation function, all of which may play a role in COVID-19 etiology. We hypothesized that the inflammatory response to severe acute respiratory syndrome coronavirus 2 (SARS-CoV-2) infection is fundamental to COVID-19 pathogenesis and that dysregulation of the immune and/or coagulation systems result in severe clinical outcomes, such as Acute respiratory distress syndrome (ARDS), coagulopathy, and septic shock, among others. Patients who died showed lower WBC and O_2_ percentages, as well as higher age, CRP, and LDH values than survivors. High-mortality-risk COVID-19 individuals may benefit from early treatment based on a comprehensive evaluation of the inflammatory response, immunologic dysfunction, and coagulopathy. As anticipated, the combination of clinical information and chest X-ray pictures aids in the accurate diagnosis of COVID-19 severity and mortality risk.

Additionally, our nomogram is applicable in a range of therapeutic contexts. To our knowledge, it outperforms other models proposed in the literature. In addition, the score of the nomogram served as a quantitative tool for identifying patients with a high risk of mortality upon admission and for guiding clinical management. COVID-based hospital admission information, 19 individuals were assigned to risk groups. In isolation centers, low-risk cases should be isolated and treated. For comprehensive care, survivors from high-risk categories should be admitted to a hospital with an isolation unit. The high-risk group is referred to the intensive care unit (ICU) for intensive treatment and assistance.

## Conclusion

The current gold standard for the identification of coronavirus illness (COVID-19) is the reverse transcription-polymerase chain reaction (RT-PCR) test, despite its drawbacks, which include a longer turnaround time, greater false-negative rates of 20–25%, and more expensive equipment. In addition, the detection of COVID-19 involves physical examinations, radiographic imaging, blood testing, and the reverse transcription polymerase chain reaction (RT-PCR) technique. Using clinical data, CT radiographic imaging, and sign symptoms, the severity of COVID-19 has been determined. There have been researches employing DNA-based methods, however, such data are not readily available, and genome sequencing investigations are computationally costly. The objective of this study is to develop a multimodal system that combines both Chest X-ray (CXR) pictures and clinical data to predict the severity of COVID-19 infection in patients. The severity classification method described by employing commonly available and less expensive radiological imaging (Chest X-rays compared to CT) and a smaller number of biomarkers that may be easily obtained from Common Blood Count tests is unquestionably a life-saving and cost-effective option. In addition, the technique improves the accuracy and dependability of the diagnosis. The proposed architecture makes use of CXR pictures and only five parameters: LDH, O_2_%, Age, WBC, and CRP, and demonstrates exceptional results for recognizing low- and high-risk COVID-19-positive individuals with extremely high sensitivity. Moreover, the proposed nomogram-based technique accurately predicts the likelihood of death among high-risk people. Our prognostic nomogram for COVID-19 patients displayed excellent discrimination and calibration based on many risk markers. Since the model utilizes CXR pictures and clinical factors, it can refute the physicians' complaints regarding the use of merely radiographic images for prognostic purposes. This approach can determine a patient's probable risk upon admission, which can considerably improve hospital resource management. Although the study used data from initial variants, the clinical biomarkers identified in this work are supported by a large pool of clinical studies conducted on other variants; as a result, we anticipate that this model will be equally applicable to Omicron and other future variants that may emerge in the upcoming winter. As a result, physicians could use this technique to make a swift and objective determination to enhance patient stratification management and possibly reduce death rates. However, this quantitative tool should be tested in large-scale prospective multicenter and multi-country trials to verify its clinical utility.

## Supplementary Information

Below is the link to the electronic supplementary material.

Supplementary file1 (DOCX 404 KB)

## Data Availability

The datasets used in this study are available from the corresponding author on reasonable request.
