# Supplementary material for: BIO-CXRNET: a robust multimodal stacking machine learning technique for mortality risk prediction of COVID-19 patients using chest X-ray images and clinical data
Source: Neural Comput Appl. 2023 May 4:1–23. Online ahead of print. doi: 10.1007/s00521-023-08606-w (PMC10157130; doi:10.1007/s00521-023-08606-w)
Supplement: Supplementary file 1 — Supplementary file1 (DOCX 404 KB) [file 521_2023_8606_MOESM1_ESM.docx]

**Supplementary Materials**


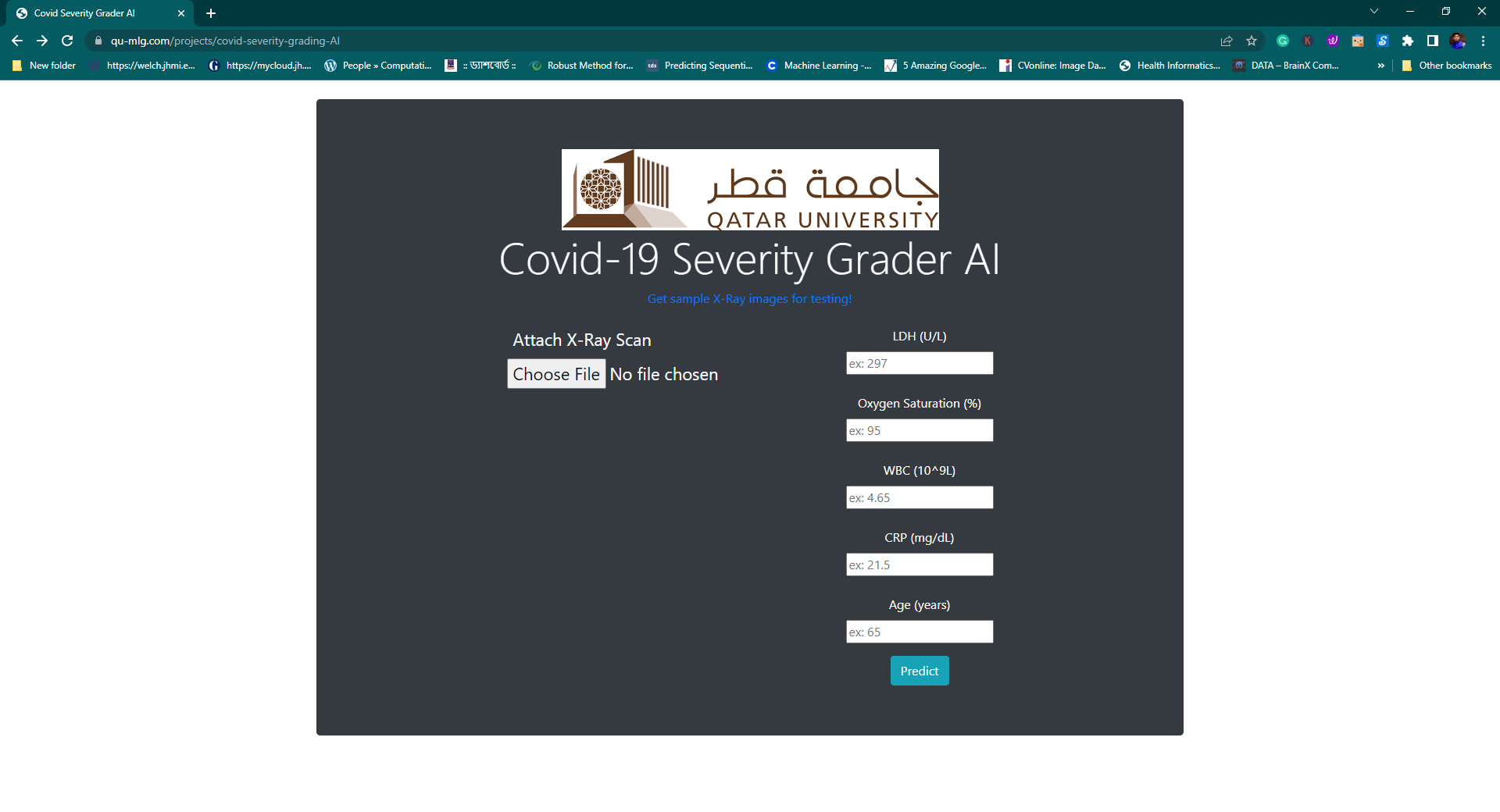


**(a)**


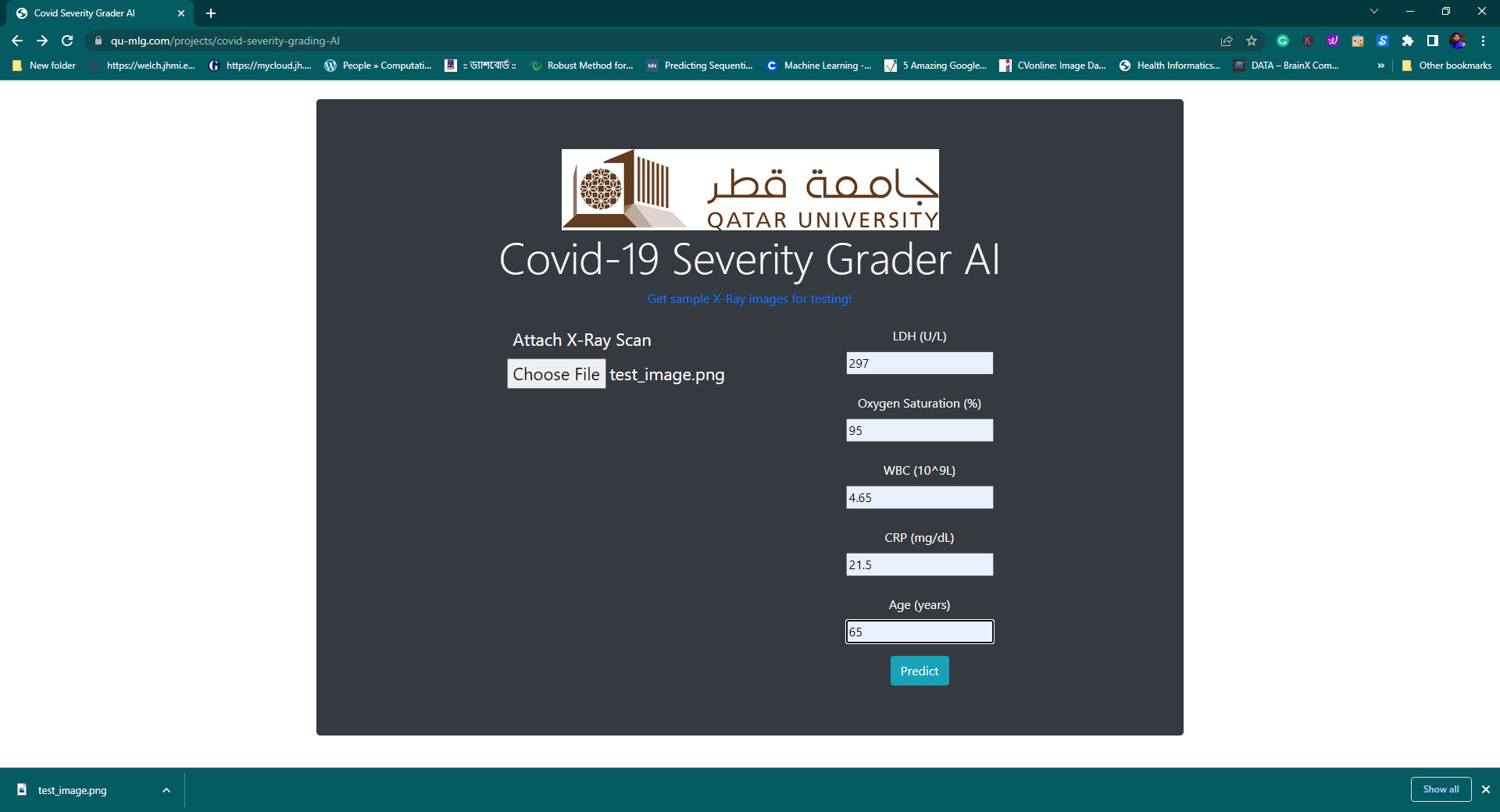


**(b)**


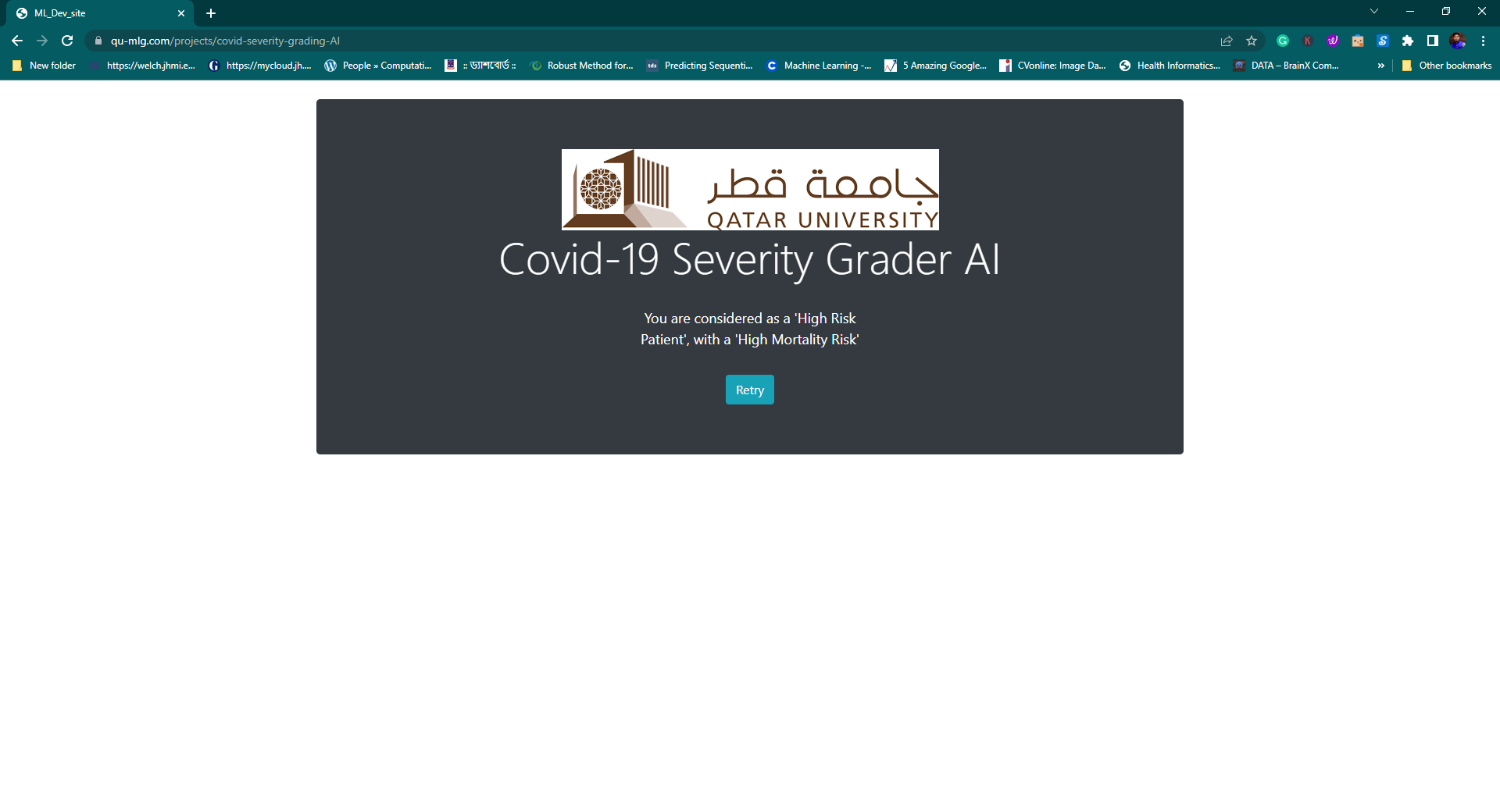


**(c)**

**Supplementary Figure 1:** Screenshot of the System (a) First page of the system where user must provide inputs, (b) User input provided, (c) Result displayed.

**Supplementary Table 1:** Finetuned parameters for the best performing classifiers.

| Classifier | Finetuned parameters |
| --- | --- |
| Linear Discriminant Analysis (LDA) | solver='eigen', shrinkage='auto', priors=None, n_components=128, store_covariance=False, tol=0.0001 |
| XGBoost (XGB) | max_depth=85, learning_rate=0.1, min_split_loss= 0.0, reg_lambda=5.93, min_child_weight= 2.77, colsample_bylevel= 0.8, colsample_bynode=0.5, alpha= 7.96, n_estimators=150 |
| Random Forest (RF) | n_estimators=75, criterion='entropy', max_depth=50, min_samples_split=2, min_samples_leaf=1, min_weight_fraction_leaf=0.0, max_features=1.0 |
| Logistic Regression (LR) | penalty='l2', dual=False, tol=0.0001, C=1.0, fit_intercept=True, intercept_scaling=1, solver='lbfgs', max_iter=100, multi_class='auto' |
| Support Vector Machine (SVM) | kernel='rbf', degree=5, gamma=auto, tol=0.001, cache_size=100, verbose=True, max_iter=-3, decision_function_shape='ovr' |
| Extra Tree (ET) | n_estimators=85, *, criterion='gini', max_depth=10, min_samples_split=2, min_samples_leaf=1, min_weight_fraction_leaf=0.0, max_features='log2' |
| K-Nearest Neighbors (KNN) | n_neighbors=10, weights='distance', algorithm='auto', leaf_size=25, p=2, metric='minkowski' |
| Gradient Boosting (GB) | oss='log_loss', learning_rate=0.01, n_estimators=110, subsample=1.0, criterion='friedman_mse', min_samples_split=2, min_samples_leaf=1, min_weight_fraction_leaf=0.0, max_depth=5 |

**Supplementary Table 2:** Performance metrics for single and multimodal data with different PCA variance for risk prediction study

| Modality | PCA variance | Accuracy | Precision | Sensitivity | F1-score | Specificity |
| --- | --- | --- | --- | --- | --- | --- |
| CXR images | 70% | 74.21 | 75.18 | 74.21 | 75.25 | 74.29 |
|  | 75% | **79.5** | **79.53** | **79.54** | **79.54** | **79.45** |
|  | 80% | 67.41 | 69.02 | 67.41 | 69.03 | 67.43 |
|  | 85% | 72.47 | 73.17 | 72.47 | 73.13 | 72.55 |
|  | 90% | 78.32 | 78.61 | 78.33 | 78.52 | 78.38 |
|  | 95% | 68.99 | 70.64 | 68.99 | 70.65 | 69.01 |
| Clinical data | 70% | 79.43 | 79.56 | 79.43 | 79.33 | 79.47 |
|  | 75% | **83.01** | **83.87** | **83.01** | **83.01** | **83.04** |
|  | 80% | 69.78 | 70.91 | 69.78 | 70.92 | 69.85 |
|  | 85% | 74.68 | 75.5 | 74.69 | 75.55 | 74.76 |
|  | 90% | 80.22 | 80.51 | 80.22 | 80.47 | 80.28 |
|  | 95% | 81.8 | 82.01 | 81.8 | 81.95 | 81.85 |
| Both  CXR images & Clinical data | 70% | 83.25 | 83.67 | 83.65 | 83.4 | 83.45 |
|  | 75% | **89.03** | **90.44** | **89.03** | **89.03** | **88.7** |
|  | 80% | 82.66 | 82.67 | 82.65 | 82.66 | 82.38 |
|  | 85% | 84.12 | 84.14 | 84.26 | 84.18 | 84.35 |
|  | 90% | 78.64 | 78.76 | 78.64 | 78.48 | 78.68 |
|  | 95% | 81.38 | 81.22 | 80.35 | 80.88 | 81.2 |

**Supplementary Table 3:** Performance metrics for single and multimodal data with different PCA variance for death prediction study

| Modality | PCA variance | Accuracy | Precision | Sensitivity | F1-score | Specificity |
| --- | --- | --- | --- | --- | --- | --- |
| CXR images | 70% | 84.81 | 84.81 | 84.81 | 84.41 | 84.81 |
|  | 75% | **86.35** | **83.22** | **86.35** | **86.35** | **87.4** |
|  | 80% | 82.91 | 83.03 | 82.91 | 82.9 | 82.94 |
|  | 85% | 81.96 | 82.03 | 81.96 | 81.78 | 81.99 |
|  | 90% | 82.12 | 82.16 | 82.12 | 81.83 | 82.14 |
|  | 95% | 80.38 | 80.54 | 80.38 | 80.38 | 80.42 |
| Clinical data | 70% | 84.34 | 84.36 | 84.34 | 84.03 | 84.35 |
|  | 75% | **91.2** | **91.25** | **91.2** | **91.2** | **91.22** |
|  | 80% | 84.65 | 84.65 | 84.65 | 83.7 | 84.6 |
|  | 85% | 83.7 | 83.7 | 83.7 | 82.65 | 83.64 |
|  | 90% | 85.44 | 85.55 | 85.44 | 84.11 | 85.34 |
|  | 95% | 84.18 | 84.21 | 84.18 | 83.91 | 84.19 |
| Both  CXR images & Clinical data | 70% | 84.18 | 84.16 | 84.18 | 83.61 | 84.16 |
|  | 75% | **92.88** | **93.37** | **92.88** | **92.88** | **92.65** |
|  | 80% | 84.81 | 84.79 | 84.81 | 84.2 | 84.8 |
|  | 85% | 84.18 | 84.23 | 84.17 | 83.98 | 84.19 |
|  | 90% | 84.18 | 84.16 | 84.17 | 83.25 | 84.13 |
|  | 95% | 83.7 | 83.72 | 83.7 | 83.38 | 83.71 |
